# CCL16 maintains stem cell-like properties in breast cancer by activating CCR2/GSK3β/β-catenin/OCT4 axis

**DOI:** 10.7150/thno.51000

**Published:** 2021-01-01

**Authors:** Wenzhi Shen, Xiaoyuan Zhang, Jiaping Tang, Zhixin Zhang, Renle Du, Dehong Luo, Xiaoran Liu, Yong Xia, Yanping Li, Shanshan Wang, Siyuan Yan, Wancai Yang, Rong Xiang, Na Luo, Yunping Luo, Jianjun Li

**Affiliations:** 1 Dept. of Pathology and Institute of Precision Medicine, Jining Medical University, Jining 272067, China.; 2 Institute of Breast Research, Jining Medical University, Jining 272067, China.; 3 Dept. of Anatomy and Histology, School of Medicine, Nankai University, Tianjin 300071, China.; 4 Dept. of Gastrointestinal Surgery, Affiliated Hospital of Jining Medical University, Jining 272029, China.; 5 Dept. of Immunology, School of Medicine, Nankai University, Tianjin 300071, China.; 6 The First People's Hospital of Zunyi, Zunyi, 563002, China.; 7 Dept. of Immunology, Institute of Basic Medical Science, Chinese Academy of Medical Science, School of Basic Medicine Peking Union Medical College, Beijing, 100005, China.

**Keywords:** CCL16, CSCs, CCR2, β-catenin, OCT4, breast cancer

## Abstract

**Rationale:** Considerable evidence suggests that breast cancer metastasis and recurrence occur due to emergence of cancer stem cells (CSCs). In our previous study, we designed a high-throughput siRNA screening platform that identifies inflammation genes involved in the regulation of cancer cell stemness. We reported that CCL16 protein decreases OCT4 expression and reduces the ALDH+ subpopulation. However, the mechanism by which CCL16 maintains stem cell-like properties remains unclear.

**Methods:** Tissue microarrays were used to evaluate CCL16 expression. Cancer stemness assays were performed in CCL16 knockdown and overexpressing cells *in vitro* and in a xenograft model *in vivo*. Human phosphokinase array, immunofluorescence and chromatin immunoprecipitation assays were performed to explore the underlying mechanism.

**Results:** We report that CCL16 was overexpressed in breast tumors and significantly correlated with clinical progression. We found that silencing CCL16 in MDA-MB-231 and BT549 cells diminished CSC properties including ALDH+ subpopulation, side population, chemo-resistance, and sphere formation. Furthermore, mice bearing CCL16-silenced MDA-MB-231 xenografts had lower tumorigenic frequency and developed smaller tumors. Exploration of the underlying mechanism found that CCL16 selects CCR2 to activate p-AKT/GSK3β signaling and facilitate β-catenin nuclear translocation. Further, CCL16 binds to the OCT4 promoter and promotes OCT4 expression. In addition, shRNAs targeting CCR2 and XAV939 targeting β-catenin abolished CCL16-mediated cancer stemness. Upstream, IL10 mediates STAT3 activation, which binds to the CCL16 promoter and enhances its expression. The STAT3-targeted inhibitor Stattic suppressed CCL16 expression* in vitro* and restrained tumor progression *in vivo*.

**Conclusions:** We identified a potential CSC regulator and suggest a novel mechanism for how CCL16 governs cancer cell stemness. We propose that CCL16 could be an effective target for breast cancer therapy.

## Introduction

Breast cancer is one of the most common malignancies in women and is among the leading causes of cancer death in women 1. Although the mortality rate of breast cancer has fallen over the past 10 years, effective treatments that prevent either recurrence, metastasis, or chemo-resistance remain lacking. Recent studies have demonstrated that tumor progression and metastasis may be linked to the emergence of cancer stem cells 2-5. However, our current understanding of which factor(s) drive the stemness signaling pathways in breast cancer remains limited even though much progress has occurred in the area of tumor progression 6-8.

Chemokine (C-C motif) ligand 16 (CCL16) is a small cytokine belonging to the CC chemokine family that is known under several pseudonyms, including liver-expressed chemokine (LEC) and monotactin-1 (MTN-1). CCL16 expression is strongly induced by IL-10, IFN-γ, and bacterial lipopolysaccharide in monocytes 9-11. CCL16 exerts its effects on cells by binding to cell surface chemokine receptors (i.e., CCR1, CCR2, CCR5, and CCR8). For example, CCL16 activates angiogenesis in vascular endothelium via CCR1 8. In addition, CCL16 is a prognostic biomarker that predicts metastasis in both triple-negative breast cancer and lung cancer 12. However, the specific role that CCL16 plays in breast cancer remains unclear.

Glycogen synthase kinase-3 beta (GSK3β) is a serine-threonine kinase belonging to the glycogen synthase kinase family. GSK3β is involved in many cellular processes like energy metabolism, inflammation, ER stress, mitochondrial dysfunction, and the apoptotic pathway 13-15. Ser9 phosphorylation of GSK3β induces a pseudo-substrate conformational change in the substrate-docking motifs of GSK3β and thereby silences GSK3β. This results in GSK3β proteasomal degradation, which has been associated with many pathological conditions including cancer. GSK3β is well-known to function through the Wnt/β-catenin signaling pathway 16. Previous reports have clearly established the role of Wnt/GSK3β/β-catenin signaling in various cancers. However, the exact factors that trigger Wnt/GSK3β/β-catenin signaling, and thereby regulate various cellular processes, have not been elucidated.

In the present study, we investigate the role and mechanism of CCL16 in the progression of breast cancer *in vitro* and *in vivo*. We demonstrate that CCL16 drives various stem cell-like properties in both MDA-MB-231 and BT549 cells *in vitro* and also in a xenograft NOD/SCID mouse model. In regard to the underlying mechanism, we demonstrate that CCL16 specifically binds to CCR2 (but no other receptors) and activates the p-AKT/GSK3β signaling pathway. Activation of the p-AKT/GSK3β signaling pathway leads to nuclear translocation of β-catenin where it binds the OCT4 promoter, which induces OCT4 expression. In addition, shRNA silencing CCR2 and XAV939 (a β-catenin inhibitor) both abolish CCL16-mediated cancer cell stemness. In regard to upstream events, we found that IL10 activates STAT3, which binds to the CCL16 promoter gene and enhances CCL16 expression, thereby increasing cancer cell stemness. Likewise, Stattic (a STAT3 phosphorylation inhibitor) deactivates STAT3 and suppresses CCL16 expression, thereby decreasing CCL16-mediated cancer cell stemness. Thus, we suggest that CCL16 may serve as an effective target in breast cancer therapy.

## Methods

### Cell culture

MDA-MB-231 breast cancer cells were cultured in L15 medium. BT594 breast cancer cells were cultured in 1640 medium. Culture media were supplemented with 10% FBS (Gibco). BT594 cells were grown at 37 ℃ in 5% CO_2_ and MDA-MB-231 cells were grown at 37 ℃ in 0% CO_2_. Cells were passaged for less than 3 months before renewal from frozen, early-passage stocks. Cells were purchased from ATCC and were tested for mycoplasma contamination.

### Vector construction

shRNA targeting human CCL16 and a scrambled control sequence are listed in **Table S1**. Palindromic DNA oligos were annealed to form a double-stranded oligo and ligated to the linearized pLV-H1-EF1α-puro (Biosettia, cat. # B19) vector to generate circled pLV-H1-shCCL16-Puro. To construct the human CCL16 overexpression plasmid, the cDNA of CCL16 was cloned using the primer pairs listed in **Table S1**. The amplified fragments were finally ligated into pLV-EF1α-MCS-IRES-Puro (Biosettia, cat. # pLV-03) expression vector to generate pLV-EF1α-CCL16-Puro.

### Quantitative RT-PCR (qPCR)

qRT-PCR was performed following a published protocol 17. The primers used in this assay are listed in **Table S1**.

### Aldehyde dehydrogenase (ALDH) assay

ALDEFLUOR kit (STEMCELL Technologies) was used to measure ALDH enzymatic activity in breast cancer cells. Briefly, 2.5 × 10^5^ cells were suspended in ALDRFLUOR assay buffer containing ALDH1 substrate and incubated for 40 min at 37 °C. Treatment with DEAB, a specific ALDH inhibitor, served as the negative control. Stained cells were analyzed on a BD FACSCalibur flow cytometer (BD Biosciences, San Jose, CA). Data analysis was performed using FlowJo software (Tree Star, Inc., Ashland, OR) 18.

### Side population assay

MDA-MB-231 and BT549 cells were harvested and resuspended in prewarmed staining buffer (PBS with 2% FBS) at a density of 1.0 × 10^6^ cells/mL. Hoechst 33342 dye was added at a final concentration of 0.008 mg/mL in the presence or absence of 0.01 mM fumitremorgin C (FTC). Subsequent steps were described previously 18, 19.

### Chemo-resistance assay

MDA-MB-231 and BT549 cells were treated with cisplatin (DDP; 0.01 mg/mL) for 24 h, then harvested and resuspended in prewarmed staining buffer (PBS with 2% FBS) at a density of 1 × 10^6^ cells/mL. Apoptotic cells were stained with propidium iodide and Annexin-V-FITC (BD Biosciences). Flow cytometry analysis was performed using a FACSCalibur cytometer (BD Biosciences), in which a minimum of 10,000 events were recorded.

### Sphere formation assay

MDA-MB-231 and BT549 cells were collected and rinsed to remove serum, then dissociated to single-cell suspensions in sphere-specific medium (STEMCELL Technologies, cat. # H4531). Cells were subsequently cultured in ultra-low attachment 24-well plates at a density of 500 cells per well.

### Immunohistochemistry

Immunohistochemistry was performed on paraffin-embedded specimens and tissue microarrays (Alenabio Company, cat. # Top 240a, cat. # MC8010) of human breast cancer. Protein expression in the tissue samples was detected using the specific antibodies listed in **Table S2**. For CCL16 tissue microarray staining, the expression levels were scored according to the percentage of CCL16+ cells and the staining intensity of each tumor specimen. Specifically, 0-25%, 26-50%, 51-75%, and 76-100% CCL16+ cells were scored as 1, 2, 3, and 4, respectively; non-significant brown, light brown, moderate brown, and deep brown staining intensities were scored as 1, 2, 3, and 4, respectively. The two scores were multiplied to get the final score 20. The other protein stainings were scored according to the percentage of protein-positive cells in each breast tissues. Images were collected using an Olympus BX51 epi-fluorescence microscope with a ×10 or ×40 objective (Olympus, Tokyo, Japan) 17.

### Immunofluorescence

Cells grown on glass slides were fixed in 4% paraformaldehyde, labeled with primary antibodies overnight at 4 °C, and then incubated with species-appropriate secondary antibodies at room temperature for 1 h. Nuclei were stained with DAPI, and images were collected using a Leica DM4000 upright microscope or a confocal fluorescence microscope (Nikon, Tokyo, Japan) 21.

### Western blot

Protein expression was detected by western blot carried out according to protocols described previously 22, 23. Primary antibodies used in this assay are listed in **Table S2**. All western blot results are provided as representative images from three independent experiments.

### Immunoprecipitation

Cell protein lysates were incubated with protein A/G agarose beads (Life technology) and antibody overnight at 4 °C. Then, the beads were centrifuged at 2500 rpm for 30 s and the pellets were carefully washed with prechilled PBS buffer. Finally, bound proteins were boiled for SDS-PAGE. Immunoprecipitates were analyzed with specific antibodies by Western blot. Antibodies used in this assay are listed in **Table S2**.

### Luciferase activity assay

Luciferase activity was determined using the Dual-Luciferase Reporter Assay System (Promega, E1910). The promoter regions were amplified by PCR and cloned into the pGL3-basic vector to create the pGL3-promoter firefly luciferase reporter plasmids. The primers used in this assay are listed in **Table S1**. For reporter assays, MDA-MB-231 and BT549 cells were transiently transfected with pGL3-promoter reporter plasmid and relative plasmids vectors. Firefly luciferase activity was normalized to *Renilla* luciferase activity for all samples to yield relative luciferase activity.

### Chromatin immunoprecipitation (ChIP) assay

The assay was performed with an EZ-Zyme Chromatin Prep Kit (Millipore) according to the manufacturer's protocol. Anti-β-catenin or anti-STAT3 antibodies were used to precipitate DNA cross-linked with β-catenin or STAT3, and normal rabbit IgG was used in parallel as a control. Enriched DNA was then used as a template to assess the binding intensity of β-catenin or STAT3 to putative binding sites in the OCT4 or CCL16 promoters, respectively 24. Primers used in this assay are listed in**Table S1**.

### Protein kinase phosphorylation assay

Protein kinase phosphorylation was assayed using a human phosphokinase array kit (R&D system, cat. # ARY003B) following the manufacturer's instructions.

### Animal studies

All *in vivo* mouse experiments were approved by the Ethics Committee of Jining Medical University. NOD/SCID female mice at 6-8 weeks of age were allocated randomly to each group (n = 8 or 10). Cells were subcutaneously injected into each mouse. Tumor size (mm^3^) was measured with calipers and calculated by the following formula: volume (mm^3^) = (width^2^ × length)/ 2. The individual measuring the tumor sizes was blinded to the treatments. Primary tumor tissues were formalin fixed, paraffin embedded, and sectioned for further analysis.

Subcutaneous tumors formed by 1 × 10^6^ MDA-MB-231 cells expressing shCtrl, shCCL16, shCCL16-OECtrl, shCCL16-CCL16R, OECtrl, OECCL16, OECCL16-shCtrl, or OECCL16-shCCR2 were dissected at 48 days after implantation.

For limited dilution transplantation, 5 × 10^5^, 1 × 10^5^, 5 × 10^4^ or 1 × 10^4^, 5 × 10^4^, 1 × 10^5^ cells were subcutaneously injected into NOD/SCID male mice. 28 days after implantation, tumor tissues were harvested.

For inhibitors treatment, NOD/SCID female mice were injected with 1 × 10^6^ MDA-MB-231 or MDA-MB-231-luci cells. 12 days after cell injection, mice were treated with XAV939 in DMSO (20 mg/kg) every 4 days or Stattic (20 mg/kg) every 3 days by intraperitoneal injection or with human IL10 (0.8 × 10^-6^ mg/mL) every 3 days by intratumor injection. Tumor volumes were calculated and bioluminescence images were captured.

### Statistical analysis

All data were analyzed using GraphPad Prism5 software (San Diego, CA, USA). Values are expressed as mean ± SEM. P-values were calculated using a two-tailed Student's t-test (two groups) or one-way ANOVA (more than 2 groups) unless otherwise noted. A value of P < 0.05 was used as the criterion for statistical significance. * Indicates a significant difference with P < 0.05, ** indicates a significant difference with P < 0.01, and *** indicates a significant difference with P < 0.001 25.

## Results

### CCL16 is up-regulated in human breast cancer tissues

In our previous study, we designed a high-throughput siRNA screening platform that identifies inflammation genes involved in the regulation of cancer cell stemness. In this platform, we used the HMLE-Snail cell line (immortalized human mammary epithelial cells by ectopic expression of Snail) as a cancer stem cell (CSC) model because of its stem cell-like properties **(Figure S1A)**. A dual-luciferase reporter construct driven by the OCT4 promoter gene was transfected into HMLE-Snail cells. We screened a siRNA library targeting 1027 human inflammation genes. After duplicated screening, we obtained 72 candidate genes that may be involved in control of OCT4 expression. Using an ALDH+ staining assay, we ascertained that knockdown of 10 genes (NFKB1, IL-1α, IL-1β, p50, p130, TRAF6, PRTN3, PDE3A, ICAM3, and CCL16) decreased the ALDH+ subpopulation in HMLE-Snail cells (**Figure S1A**) 18. Of these 10 genes, previous reports have demonstrated that CCL16 shows little association with CSC properties. Therefore, we decided to focus our attention on CCL16. In order to confirm our screening results, we knocked down CCL16 expression in HMLE-Snail cells. We found that knockdown of CCL16 expression decreases OCT4 expression (**Figure S1B**) and decreases OCT4 promoter gene activity (**Figure S1C**), as assessed by qPCR and Western blot (**Figure S1D**).

**Figure 1:**
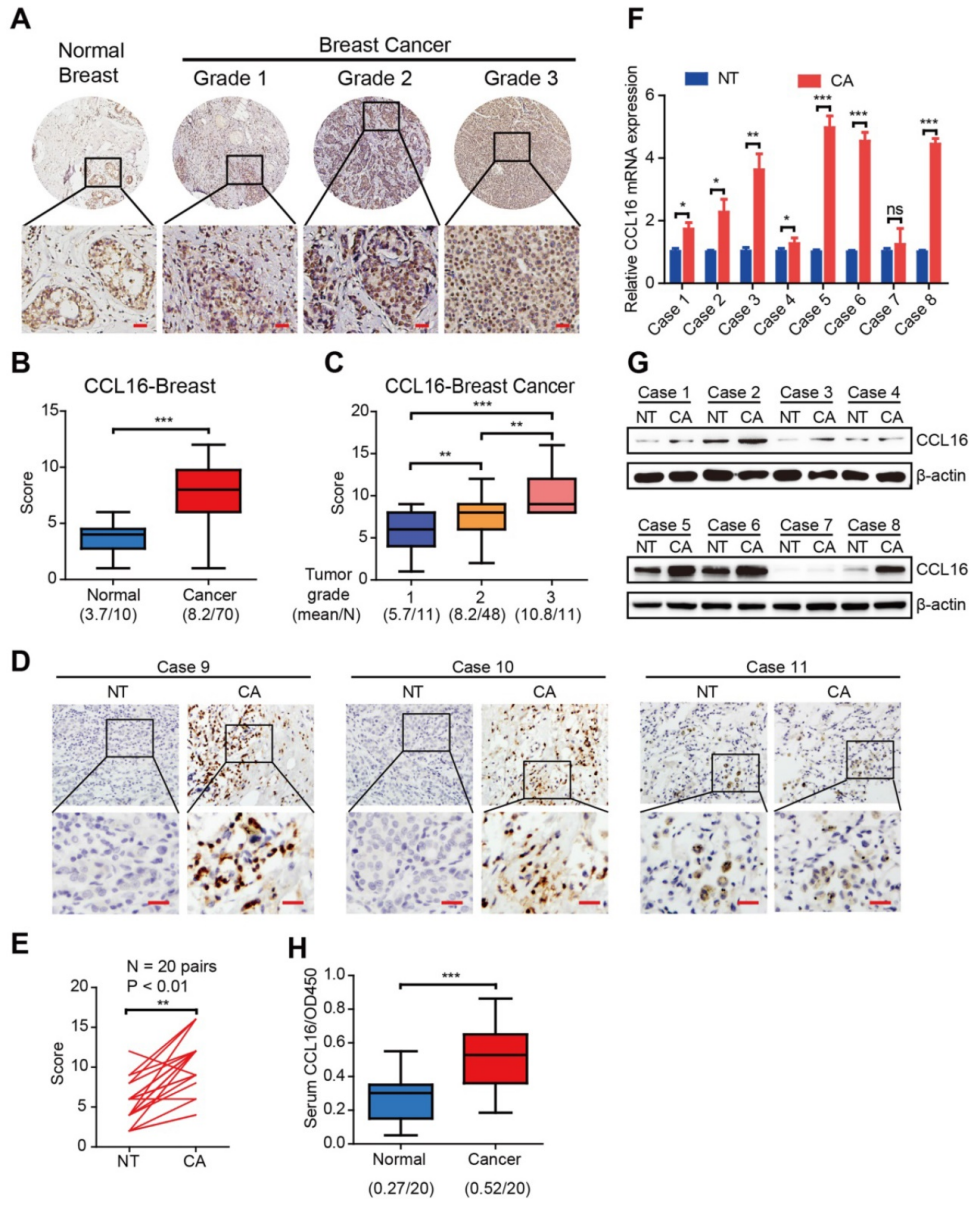
** The expression and clinical significance of CCL16 in patients with breast cancer. A. Representative immunohistochemistry of CCL16 in a human breast cancer tissue array.** This figure shows qualitatively that CCL16 immunostaining increases in breast cancer tissue versus normal breast tissue. Scale bar: 50 μm **B. Quantification of CCL16 immunohistochemistry in a human breast cancer tissue array.** This figure shows quantitatively that CCL16 immunostaining increases in breast cancer tissue versus normal breast tissue. **C. Quantification of CCL16 immunostaining and pathological grades.** This figure shows a positive correlation between CCL16 immunostaining and pathological grades 1, 2, and 3. **D. & E. Representative immunohistochemistry of CCL16 and quantitative analysis in 20 pairs breast cancer tissue and normal adjacent tissue.** These figures show that both qualitatively and quantitatively CCL16 immunostaining increases in breast cancer tissue versus normal adjacent tissue. Scale bar: 50 um **F. & G. Representative qRT-PCR and Western blot analysis of 8 pairs of fresh breast cancer tissue and normal adjacent tissue.** These figures show that CCL16 mRNA expression increases in breast cancer tissue versus normal adjacent tissue. **H. Measurement of serum CCL16 levels in 20 pairs of breast cancer patients and normal patients.** This figure shows that serum CCL16 levels significantly increase in breast cancer patient serum (n = 20) versus normal patient serum (n = 20).

To investigate the CCL16 expression pattern in human breast cancers, we performed immunohistochemistry using a CCL16-specific antibody in breast cancer tissue microarrays containing 80 biopsies (70 breast cancer tissues and 10 normal breast tissues). The results show that CCL16 immunostaining is stronger in breast cancer tissue versus normal breast tissue (**Figure 1A-B**). Since the clinical pathological grade of a tumor closely correlates to tumor malignancy/differentiation, we explored the correlation between CCL16 immunostaining and pathological grade (grades 1, 2, and 3). We found a positive correlation between CCL16 immunostaining and pathological grades 1, 2, and 3 for the biopsies (**Figure 1C**). To further confirm this finding, we investigated CCL16 immunostaining of tissue slides containing 20 pairs of breast cancer tissue and adjacent normal breast tissue. The results show that CCL16 immunostaining is stronger in breast cancer tissue versus adjacent normal breast tissue (**Figure 1D-E**). Moreover, we confirmed the stronger CCL16 immunostaining in 8 pairs of fresh breast cancer tissue and adjacent normal breast tissue by qRT-PCR and Western blot (**Figure 1F-G**). In addition, we measured serum CCL16 (sCCL16) levels in serum from patients with breast cancer and healthy subjects (n = 20). We found elevated sCCL16 levels in serum from patients with breast cancer versus healthy subjects (**Figure 1H**). Together, the above findings suggest that CCL16 acts as a potential cancer stemness mediator and demonstrate up-regulation of CCL16 in human breast cancer tissues.

### CCL16 plays a vital role in the maintenance of cancer stem cell-like identity *in vitro*

**Figure 2:**
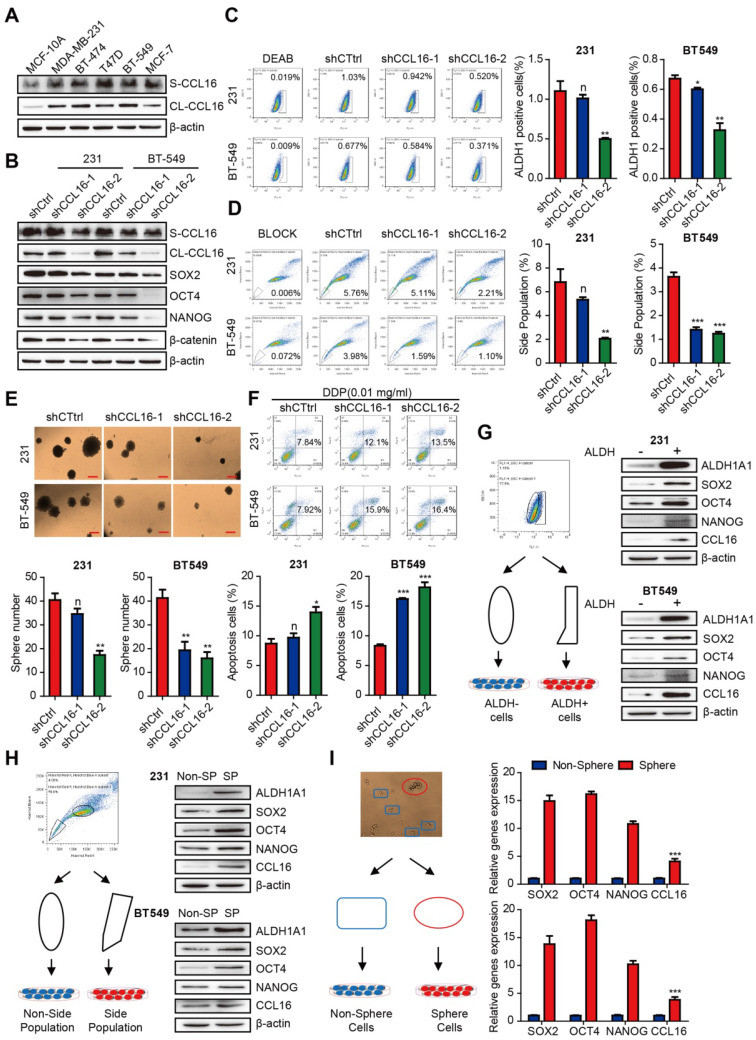
** CCL16 promotes the CSC-like characteristics in breast cancer cells. A. Western blot analysis of CCL16 expression in breast cancer cell lines and normal breast cell lines.** This figure shows that CCL16 increases especially in the MDA-MB-231 and BT-549 cell lines versus normal breast cell lines. **B. Western blot analysis.** This figure shows that SOX2, OCT4, NANOG and β-catenin expression levels decrease in shCCL16-1 and shCCL16-2 MDA-MB-231 and BT549 cells versus shCtrl cells (controls). **C. ALDH staining assay.** This figure shows that the ALDH+ sub-population decreases in shCCL16-1 and shCCL16-2 MDA-MB-231 and BT549 cells versus shCtrl cells (controls). **D. Side population assay.** This figure shows that the side-population decreases in shCCL16-1 and shCCL16-2 MDA-MB-231 and BT549 cells versus shCtrl cells (controls). **E. Sphere formation assay.** This figure shows that sphere formation decreases in shCCL16-1 and shCCL16-2 MDA-MB-231 and BT549 cells versus shCtrl cells (controls). **F. Chemo-resistance assay.** This figure shows that apoptosis increases and thereby reduces cisplatin-resistance in shCCL16-1 and shCCL16-2 MDA-MB-231 and BT549 cells versus shCtrl cells (controls). **G. Flow cytometry cell sorting by ALDH staining (left side) and Western blot analysis (right side).** This figure shows that expression levels of stemness markers (ALDH1A1, SOX2, OCT4, and NANOG) and CCL16 increase in ALDH+ versus ALDH- MDA-MB-231 and BT549 cells. **H. Flow cytometry cell sorting by side population (left side) and Western blot analysis (right side).** This figure shows that expression levels of stemness markers (ALDH1A1, SOX2, OCT4, and NANOG) and CCL16 increase in the CSC-like side population versus non-side population MDA-MB-231 and BT549 cells. **I. Flow cytometry cell sorting by sphere formation (left side) and qPCR analysis (right side).** This figure shows that mRNA expression levels of stemness markers (ALDH1A1, SOX2, OCT4, and NANOG) and CCL16 increase in CSC-like sphere versus non-sphere MDA-MB-231 and BT549 cells.

To verify the observations in clinical samples, we studied the *in vitro* CCL16 expression by Western blot analysis of sCCL16 and total CCL16 protein in breast cancer cell lines and normal breast cell lines. The results show that CCL16 expression is higher in breast cancer cell lines (especially in MDA-MB-231 and BT549 cell lines) versus normal breast cell lines (**Figure 2A**).

To test whether CCL16 regulates the stemness of breast cancer cells, we examined the mechanism by which CCL16 regulates CSCs using various experimental approaches. We first knocked down CCL16 expression by stable expression of two CCL16 shRNAs (shRNA1, shRNA2) and shCtrl (control) in MDA-MB-231 and BT549 cells. We found that the expression levels of stemness markers (i.e., SOX2, OCT4, NANOG, and β-catenin) are consistently lower in shCCL16 versus shCtrl cells (**Figure 2B**). We next analyzed changes in the ALDH+ subpopulation using an ALDH staining assay in shCCL16 and shCtrl MDA-MB-231 and BT549 cells. Our results show that the ALDH+ subpopulation is lower in shCCL16 versus shCtrl cells (**Figure 2C**). Furthermore, we examined changes in the side population (SP) of shCCL16 and shCtrl MDA-MB-231 and BT549 cells. Our results indicate that the SP is lower in shCCL16 versus shCtrl cells (**Figure 2D**). In addition, we studied the changes in sphere formation of shCCL16 and shCtrl MDA-MB-231 and BT549 cells. Our results show that sphere formation is lower in shCCL16 versus shCtrl cells (**Figure 2E**). We also investigated apoptosis after cisplatin (DDP) treatment in shCCL16 and shCtrl MDA-MB-231 and BT549 cells. We found that apoptosis is increased and cisplatin resistance reduced in shCCL16 versus shCtrl cells (**Figure 2F**).

To further confirm the role of CCL16 in the maintenance of CSC properties, we examined the expression levels of stemness markers in CSC-like ALDH+ and ALDH- MDA-MB-231 and BT549 cells separated by FACS. We observed that the expression levels of ALDH1A1, SOX2, OCT4, NANOG, and CCL16 are higher in CSC-like ALDH+ cells versus ALDH- cells (**Figure 2G**).

We also examined the expression levels of stemness markers in CSC-like SP and non-SP MDA-MB-231 and BT549 cells separated by FACS. We observed that the expression levels of ALDH1A1, SOX2, OCT4, NANOG, and CCL16 are higher in CSC-like SP cells versus non-SP cells (**Figure 2H**). We also examined the mRNA expression levels of SOX2, OCT4, NANOG, and CCL16 in CSC-like sphere and non-sphere MDA-MB-231 and BT549 cells. Our results indicate that the mRNA expression levels of SOX2, OCT4, NANOG, and CCL16 are higher in CSC-like sphere cells versus non-sphere cells (**Figure 2I**).

**Figure 3:**
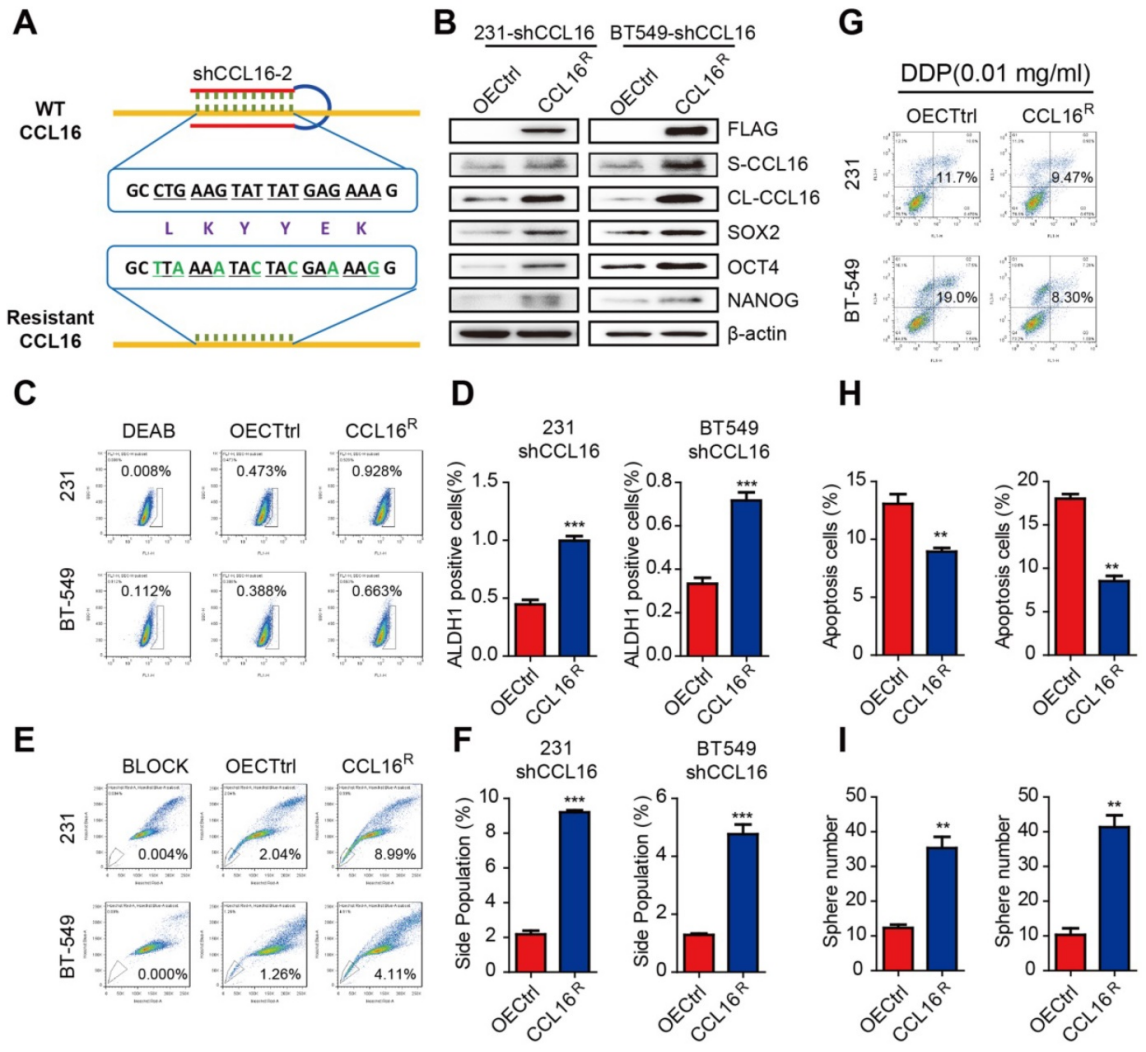
** CCL16^R^ ectopic expression rescues CCL16-mediated CSC-like properties of breast cancer cells. A. Sequence of the shRNA-resistant CCL16 (CCL16^R^). B. Western blot analysis.** This figure shows that the expression of stemness markers (SOX2, OCT4, and NANOG) increase in MDA-MB-231-shCCL16 and BT549-shCCL16 cells versus OECtrl cells (controls). In addition, FLAG, S-CCL16, CL-CCL16, and β-catenin demonstrate stable expression. Check this figure legend against what is said in the Results section. **C. & D. Flow cytometry: ALDH staining and quantitation.** These figures show that the ALDH+ sub-population increases in MDA-MB-231-shCCL16 and BT549-shCCL16 cells versus OECtrl cells (controls).** E. & F. Flow cytometry: side-population and quantitation.** These figures show that the side-population increases in MDA-MB-231-shCCL16 and BT549-shCCL16 cells versus OECtrl cells (controls).** G. & H. Chemo-resistance assay and quantification.** These figures show that apoptosis decreases and thereby increases cisplatin-resistance in MDA-MB-231-shCCL16 and BT549-shCCL16 cells versus OECtrl cells (controls).** I. Sphere formation assay.** This figure shows that sphere formation increases in MDA-MB-231-shCCL16 and BT549-shCCL16 cells versus OECtrl cells (controls).

To further evaluate the role of CCL16 in cancer cell stemness, we constructed an shRNA-resistant CCL16 (CCL16^R^) and stably expressed CCL16^R^ in MDA-MB-231-shCCL16 and BT549-shCCL16 cells (**Figure 3A**). We then examined the expression levels of stemness markers in CCL16^R^ and OECtrl (control) MDA-MB-231-shCCL16 and BT549-shCCL16 cells. We observed that the expression levels of SOX2, OCT4, and NANOG are higher in the CCL16^R^ versus OECtrl cells (**Figure 3B**). Moreover, we found that the ALDH+ subpopulation (**Figure 3C-D**), side population (**Figure 3E-F**), chemo-resistance to DDP (**Figure 3G-H**), and sphere formation (**Figure 3I**) are higher in the CCL16^R^ cells versus OECtrl cells. The above findings suggest that CCL16 plays a vital role in the maintenance of cancer stem cell-like identity.

### CCL16 enhances CSC-like characteristics in breast cancer cells *in vivo*

**Figure 4:**
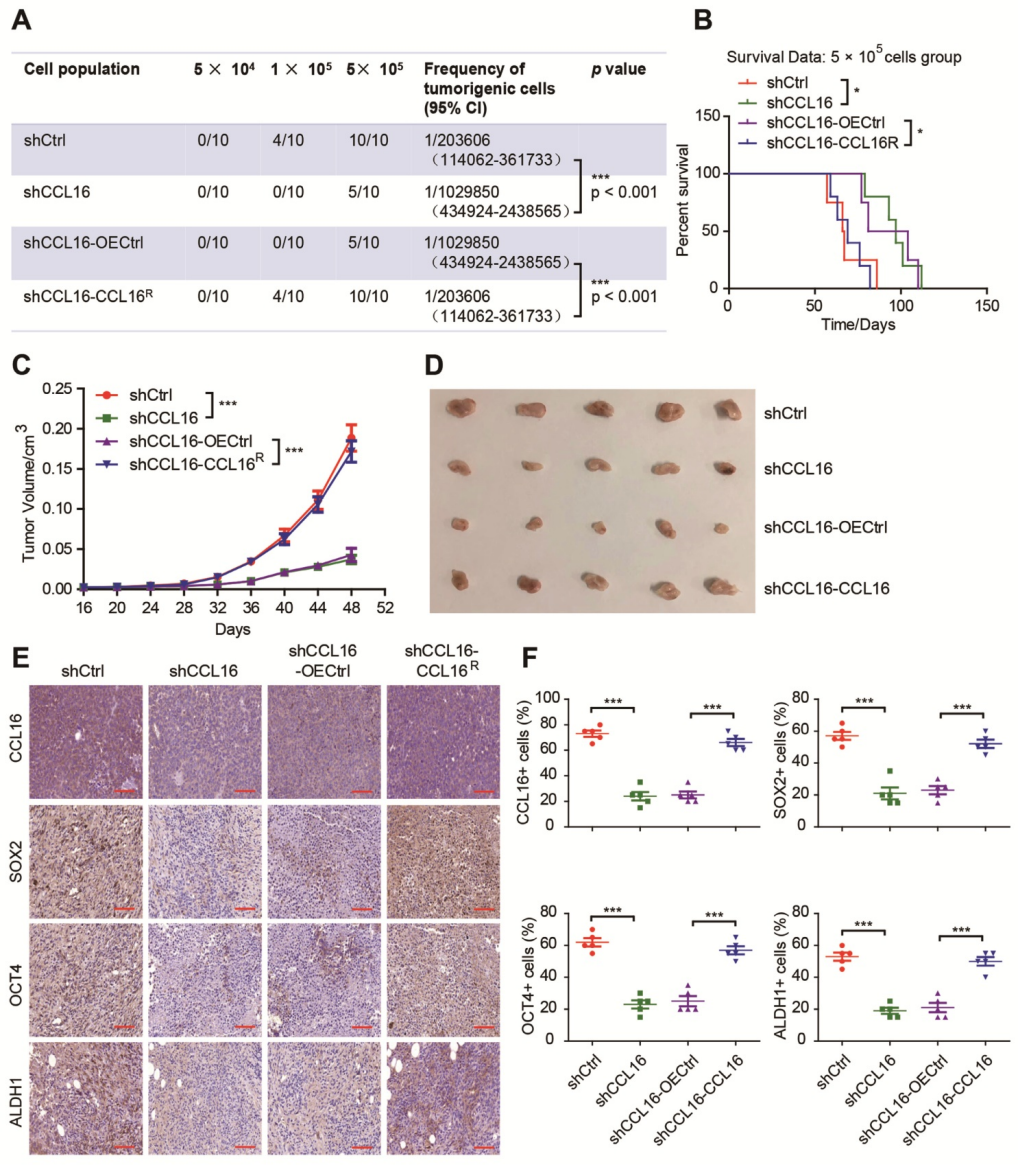
** CCL16 enhances the CSC-like characteristics of breast cancer *in vivo*. A. Limited dilution xenograft assay.** This figure shows that tumor initiation decreases in shCCL16 (CCL16 knockdown) versus shCtrl (control) 1 **×** 10^5^ MDA-MB-231 cells xenografted in NOD/SCID mice. In addition, this decrease in tumor initiation is reversed in shCCL16-CCL16^R^ (CCL16^R^ ectopic expression) versus shCCL16-OECtrl (control) MDA-MB-231 cells. Subcutaneous tumors were harvested on the 28th-day post grafting (n = 10). CI means confidence interval. **B. Survival curve of mice in the 5 × 10^5^ cell group.** This figure shows mouse survival time increases in shCCL16 (CCL16 knockdown) versus shCtrl (control) MDA-MB-231 cells xenografted in NOD/SCID mice. The increased survival time was reversed in shCCL16-CCL16^R^ (CCL16^R^ ectopic expression) versus shCCL16-OECtrl (control) MDA-MB-231 cells xenografted in NOD/SCID mice. **C. & D. Tumor growth curve and representative dissected tumors.** These figures show that both tumor growth and tumor volume decrease in shCCL16 (CCL16 knockdown) versus shCtrl (control) MDA-MB-231 cells xenografted in NOD/SCID mice. The decreases in tumor growth and tumor volume were reversed in shCCL16-CCL16^R^ (CCL16^R^ ectopic expression) versus shCCL16-OECtrl (control) MDA-MB-231 cells xenografted in NOD/SCID mice. **E. & F. Immunohistochemistry and quantification.** These figures show that immunostaining levels of CCL16, SOX2, OCT4 and ALDH1 decrease in shCCL16 (CCL16 knockdown) versus shCtrl (control) MDA-MB-231 cells. The decreases in immunostaining were reversed in shCCL16-CCL16^R^ (CCL16^R^ ectopic expression) versus shCCL16-OECtrl (control) MDA-MB-231 cells. 40X mag; Scale bar: 50 μm

To gain insight into the importance of CCL16 in the promotion of stemness in breast cancer cells *in vivo*, we performed xenograft experiments using a limited dilution xenograft assay of MDA-MB-231 cells. We found that NOD/SCID mice inoculated with 1 × 10^5^ CCL16-knockdown cells had dramatically decreased incidence of tumor initiation than mice inoculated with control cells. This decreased incidence was reversed by an increase in CCL16 levels (**Figure 4A**). Furthermore, the survival data of mice inoculated with 5 × 10^5^ CCL16-knockdown cells versus shCtrl cells show that CCL16 knockdown prolonged mouse survival. This prolongation in survival time was reversed by ectopic expression of CCL16^R^ (**Figure 4B**). In addition, knockdown of CCL16 significantly reduced both tumor growth and tumor volume. Both tumor growth and tumor volume were rescued by ectopic expression of CCL16^R^ (**Figure 4C-D**). Finally, OCT4, SOX2, and ALDH1 immunostaining levels were consistently lower in CCL16-knockdown versus shCtrl tumors. This decrease was reversed by ectopic expression of CCL16^R^ (**Figure 4E-F**). The above results collectively suggest a role for CCL16 in enhancing CSC-like characteristics in breast cancer cells *in vivo*.

### CCL16 mediates GSK3β/β-catenin signaling activation that regulates OCT4 expression *in vitro*

**Figure 5:**
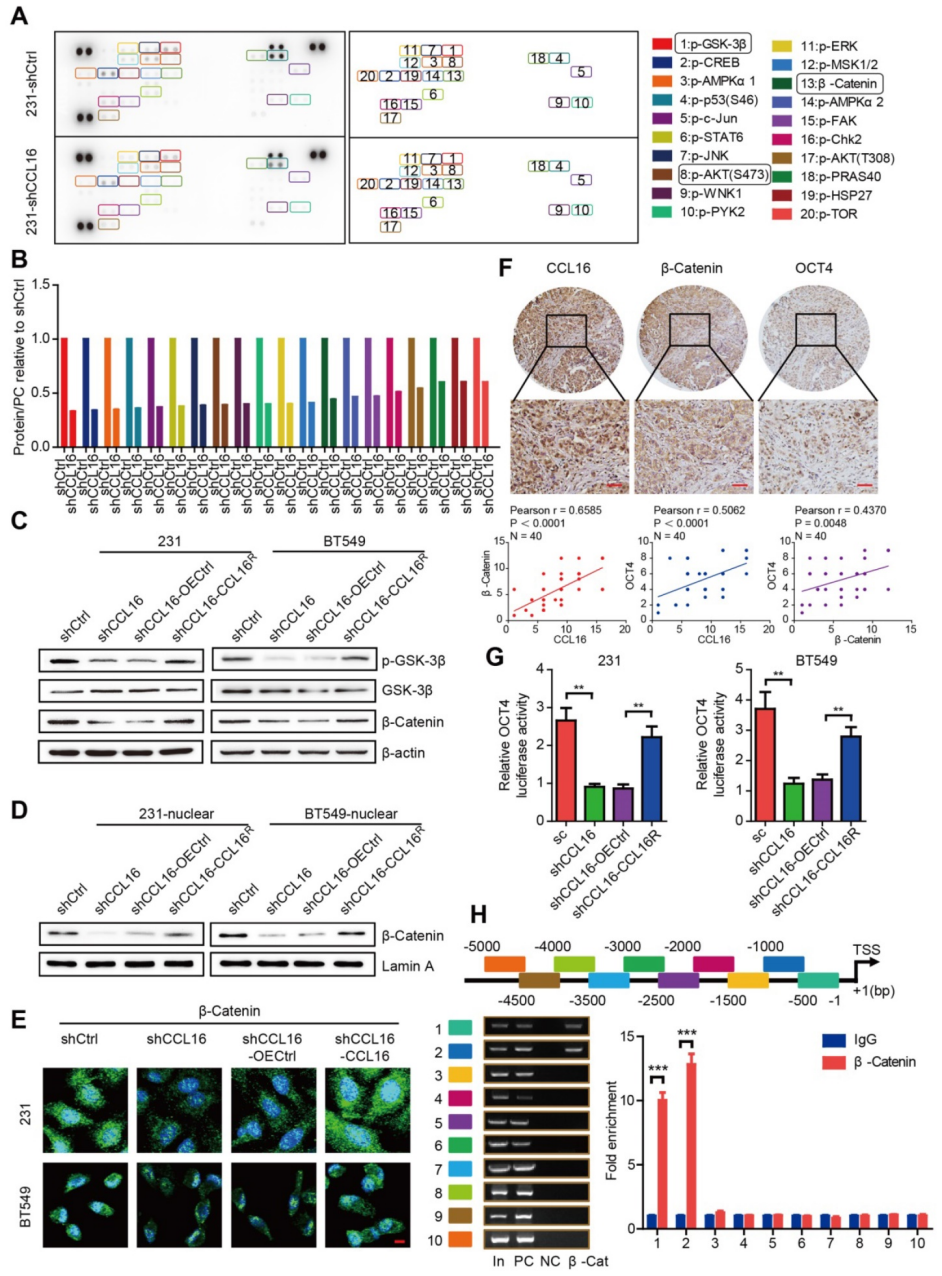
** CCL16 mediates GSK3β/β-catenin signaling activation to regulate OCT4 expression *in vitro*. A & B. Human phosphokinase array and quantification.** These figures show that the expression of 19 phosphokinases (especially p-GSK-3β) and 1 protein (i.e., β-catenin) decreases in shCCL16 (CCL16 knockdown) versus shCtrl (control) MDA-MB-231 cells. **C.** Western blot analysis. This figure shows that the expression of p-GSK3β, GSK3β, and β-catenin decreases in shCCL16 (CCL16 knockdown) versus shCtrl (control) MDA-MB-231 and BT549 cells. The decreases in expression were reversed in shCCL16-CCL16^R^ (CCL16^R^ ectopic expression) versus shCCL16-OECtrl (control) MDA-MB-231 and BT549 cells. **D. Western blot analysis.** This figure shows that the expression of β-catenin in the nucleus decreases in shCCL16 (CCL16 knockdown) versus shCtrl (control) MDA-MB-231 and BT549 cells. The decrease in expression was reversed in shCCL16-CCL16^R^ (CCL16^R^ ectopic expression) versus shCCL16-OECtrl (control) MDA-MB-231 and BT549 cells. **E. Immunofluorescence staining.** This figure shows that β-catenin immunostaining in the nucleus decreases in shCCL16 (CCL16 knockdown) versus shCtrl (control) MDA-MB-231 and BT549 cells. The decrease in immunostaining was reversed in shCCL16-CCL16^R^ (CCL16^R^ ectopic expression) versus shCCL16-OECtrl (control) MDA-MB-231 and BT549 cells. **F. Representative immunohistochemistry and correlation graphs of a human breast cancer tissue array.** This figure shows the immunostaining of CCL16, β-catenin, and OCT4 in human breast cancer tissue. In addition, the correlation graphs show a positive correlation between CCL16 and β-catenin immunostaining along with a positive correlation between CCL16 and OCT4 immunostaining. Scale bar: 50 μm **G. Dual-luciferase assay.** This figure shows that the activity of the OCT4 promoter decreases in shCCL16 (CCL16 knockdown) versus sc (control) MDA-MB-231 and BT549 cells. The decrease in activity was reversed in shCCL16-CCL16^R^ (CCL16^R^ ectopic expression) versus shCCL16-OECtrl (control) MDA-MB-231 and BT549 cells. **H.** CHIP-qPCR analysis. This figure shows that the OCT4 promoter gene contains β-catenin binding sites at -5 kb to -1 bp.

To investigate the underlying mechanism of CCL16 mediation of stemness, we used a human phosphokinase array that contains 43 kinase phosphorylation sites and 2 related total proteins in MDA-MB-231-shCCL16 and MDA-MB-231-shCtrl cells. Our results show that CCL16 knockdown reduced the expression of 19 phosphokinases (especially p-GSK-3β) and 1 total protein (β-catenin) (**Figure 5A-B**). Based on this finding, we focused our attention on the GSK-3β/β-catenin signaling pathway using a variety of methods. Using Western blot, we observed that CCL16 knockdown consistently decreased p-GSK-3β and β-catenin expression levels in MDA-MB-231 and BT549 cells. This decreased expression was reversed by ectopic expression of CCL16^R^ (**Figure 5C**). Furthermore, using nuclear extraction and immunofluorescence, we found that CCL16 knockdown decreased β-catenin expression levels in the nuclei of MDA-MB-231 and BT549 cells. This decreased expression in the nucleus was reversed by ectopic expression of CCL16^R^ (**Figure 5D-E**).

In order to confirm the correlation between CCL16 and β-catenin/OCT4, we studied the expression levels of CCL16, β-catenin, and OCT4 using immunohistochemistry in 40 human breast cancer samples. Our results reveal that at low (**Figure S2A**), moderate (**Figure S2B**), and high (**Figure 5F**) CCL16 expression levels, both β-catenin and OCT4 are also expressed. A positive correlation exists that demonstrates the co-expression of CCL16 with β-catenin and the co-expression of CCL16 with OCT4.

We next screened CCL16 using our high-throughput screening system, which suggested that CCL16 may mediate β-catenin nuclear translocation that then regulates OCT4 expression. To further study this suggestion, we measured OCT4 promoter activity in MDA-MB-231 and BT549 cells using a dual-luciferase assay. We found that CCL16 knockdown reduced OCT4 promoter activity, which is consistent with our screening results, and that CCL16^R^ ectopic expression rescued this reduction in OCT4 promoter activity (**Figure 5G**). To better understand the regulatory role of β-catenin on the OCT4 promoter, we explored whether the human OCT4 promoter gene contains β-catenin binding sites. Using a ChIP-qPCR assay, we scanned the human OCT4 promoter gene from -5 kb to the transcription start site (+1). The scan revealed that the OCT4 promoter gene contains β-catenin binding sites at -5 kb to -1 bp (**Figure 5H**). The above results collectively suggest that CCL16 mediates GSK3β/β-catenin signaling activation that promotes β-catenin nuclear translocation, which then regulates OCT4 expression.

### CCL16 binds CCR2, which causes p-AKT activation that further mediates GSK3β/β-catenin/OCT4 signaling activation *in vitro*

**Figure 6:**
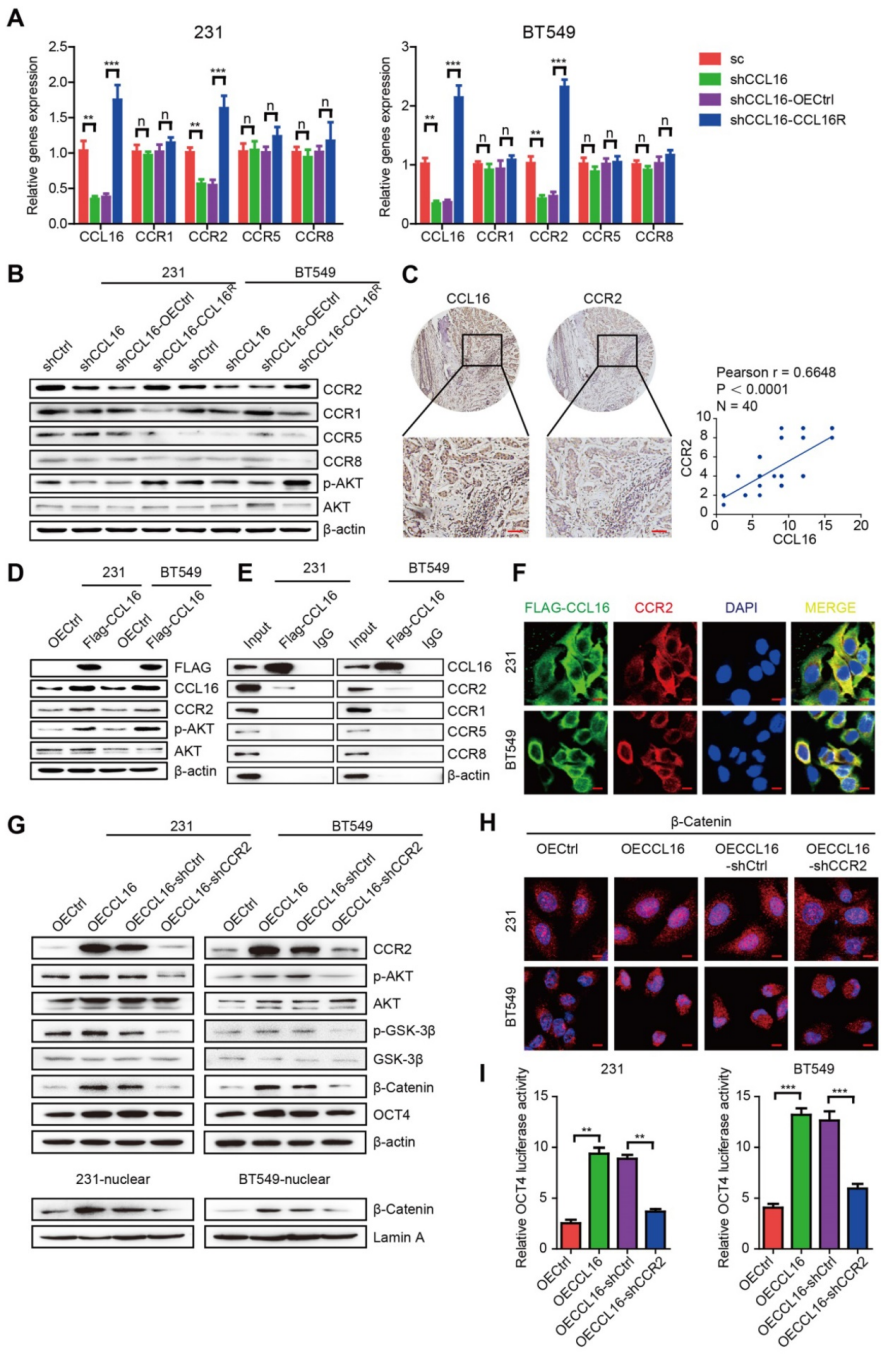
** CCL16 binds CCR2 that causes p-AKT activation which further mediates GSK3β/β-catenin/OCT4 signaling activation *in vitro.* A. QPCR analysis.** This figure shows that mRNA expression of CCR2 decreases in shCCL16 (CCL16 knockdown) versus shCtrl (control) MDA-MB-231 and BT549 cells. This decrease in mRNA expression was reversed in shCCL16-CCL16^R^ (CCL16^R^ ectopic expression) versus shCCL16-OECtrl (control) MDA-MB-231 and BT549 cells. The mRNA expression of CCR1, CCR5, and CCR8 remains unchanged. **B. Western blot analysis.** This figure shows that protein expression of CCR2 and pAKT decreases in shCCL16 (CCL16 knockdown) versus shCtrl (control) MDA-MB-231 and BT549 cells. These decreases in protein expression were reversed in shCCL16-CCL16^R^ (CCL16^R^ ectopic expression) versus shCCL16-OECtrl (control) MDA-MB-231 and BT549 cells. The protein expression of CCR1, CCR5, and CCR8 remains unchanged. **C. Representative immunohistochemistry and correlation graphs of a human breast cancer tissue array.** This figure shows the immunostaining of CCL16 and CCR2 in human breast cancer tissue. In addition, the correlation graph shows a positive correlation between CCL16 and CCR2 immunostaining Scale bar: 50 μm **D. Western blot analysis.** This figure shows that the expression of p-AKT and CCR2 increases in FLAG-CCL16 versus OECtrl (control) MDA-MB-231 and BT549 cells. **E. Immunoprecipitation-immunoblot analysis.** This figure shows that only CCR2 is pulled down by FLAG-CCL16 antibody versus IgG (control) in MDA-MB-231 and BT549 cells. **F. Double immunofluorescence staining.** This figure shows that FLAG-CCL16 and CCR2 co-localize in MDA-MB-231 and BT549 cells. Scale bar: 10um **G. Western blot analysis (upper panel, lower panel).** This figure (upper panel) shows that the expression of CCR2, p-AKT, p-GSK3β, β-catenin, and OCT4 increases in OECCL16 (CCL16 ectopic expression) versus OECtrl (control) MDA-MB-231 and BT549 cells. In addition, these increases in protein expression were reversed in OECCL16-shCCR2 (CCR2 knockdown) versus OECCL16-shCtrl (control) MDA-MB-231 and BT549 cells. This figure (lower panel) shows that expression of β-catenin in the nucleus increases in OECCL16 (CCL16 ectopic expression) versus OECtrl (control) MDA-MB-231 and BT549 cells. In addition, this increase in nuclear β-catenin expression is reversed in OECCL16-shCCR2 (CCR2 knockdown) versus OECCL16-shCtrl (control) MDA-MB-231 and BT549 cells. **H. Immunofluorescence staining**. This figure shows that immunostaining of β-catenin in the nucleus increases in OECCL16 (CCL16 ectopic expression) versus OECtrl (control) MDA-MB-231 and BT549 cells. In addition, this increase in immunostaining of β-catenin in the nucleus is reversed in OECCL16-shCCR2 (CCR2 knockdown) versus OECCL16-shCtrl (control) MDA-MB-231 and BT549 cells. **I. Dual-luciferase assay.** This figure shows that OCT4 promoter activity increases OECCL16 (CCL16 ectopic expression) versus OECtrl (control) MDA-MB-231 and BT549 cells. In addition, this increase in OCT4 promoter activity is reversed in OECCL16-shCCR2 (CCR2 knockdown) versus OECCL16-shCtrl (control) MDA-MB-231 and BT549 cells.

Previous reports have established that CCL16 activity depends upon its interaction with cell surface chemokine receptors (CCRs), mainly CCR1, CCR2, CCR5, and CCR8. To confirm which CCR is responsible for CCL16-mediated downstream GSK3β/β-catenin/OCT4 signaling activation, we studied the mRNA and protein expressions of CCRs using qPCR and Western blot in MDA-MB-231 and BT549 cells. We found that CCL16 knockdown decreased only CCR2 expression (both mRNA and protein) and that CCL16^R^ ectopic expression reversed this decrease (**Figure 6A-B**). We also studied the correlation between CCL16 and CCR2 using immunohistochemistry in 40 human breast cancer tissues. The results show a positive correlation between CCL16 and CCR2 immunostaining (**Figure 6C**).

Since AKT phosphorylation is a classic downstream target of CCR2, we next checked p-AKT and CCR2 expression in MDA-MB-231 and BT549 cells. We found that CCL16 knockdown decreased the expression of p-AKT and CCR2 and that CCL16^R^ ectopic expression rescued the expression of p-AKT and CCR2 (**Figure 6B**).

These findings closely correlate with our results obtained using the phosphokinase array (**Figure 5A**). To further verify the interaction between CCL16 and CCR2, we ectopically overexpressed FLAG-labeled CCL16 in MDA-MB-231 and BT549 cells. We found that FLAG-labeled CCL16 increased the expression of p-AKT and CCR2 and that FLAG-CCL16 antibody pulled down only CCR2 (**Figure 6D-E**). In order to morphologically reinforce the above findings, we immunostained the FLAG-CCL16 and CCR2 using double immunofluorescence in MDA-MB-231 and BT549 cells. Our results show that FLAG-CCL16 and CCR2 co-localize (**Figure 6F**).

To explore the role of CCR2 in CCL16-mediated CSC-like identity maintenance, we studied the ALDH+ sub-population, side population, and sphere formation in CCR2-knockdown MDA-MB-231-OECCL16 and BT549-OECCL16 cells. Our results show that CCL16 ectopic expression increased the ALDH+ sub-population, side population, and sphere numbers, which were decreased by CCR2 knockdown (**Figure S3A-F**). Moreover, we found that CCL16 ectopic expression increased expression of CCR2, p-AKT, p-GSK3β, β-catenin, and OCT4, which were prevented by CCR2 knockdown (**Figure 6G**, upper panel). We also noticed that CCL16 ectopic expression increased the expression of β-catenin in the nucleus and that CCR2 knockdown prevented this increase (**Figure 6G**, lower panel, and** Figure 6H**). Finally, using a dual-luciferase assay, we found that CCL16 ectopic expression increased OCT4 promoter activity, which was prevented by CCR2 knockdown (**Figure 6I**).

The above results collectively imply that CCL16 binds CCR2, which causes p-AKT activation that further mediates GSK3β/β-catenin/OCT4 signaling activation *in vitro*.

### CCR2 plays an indispensable role in CCL16-mediated p-AKT/GSK3β/β-catenin/OCT4 signaling activation and CSC-like identity maintenance *in vivo*

**Figure 7:**
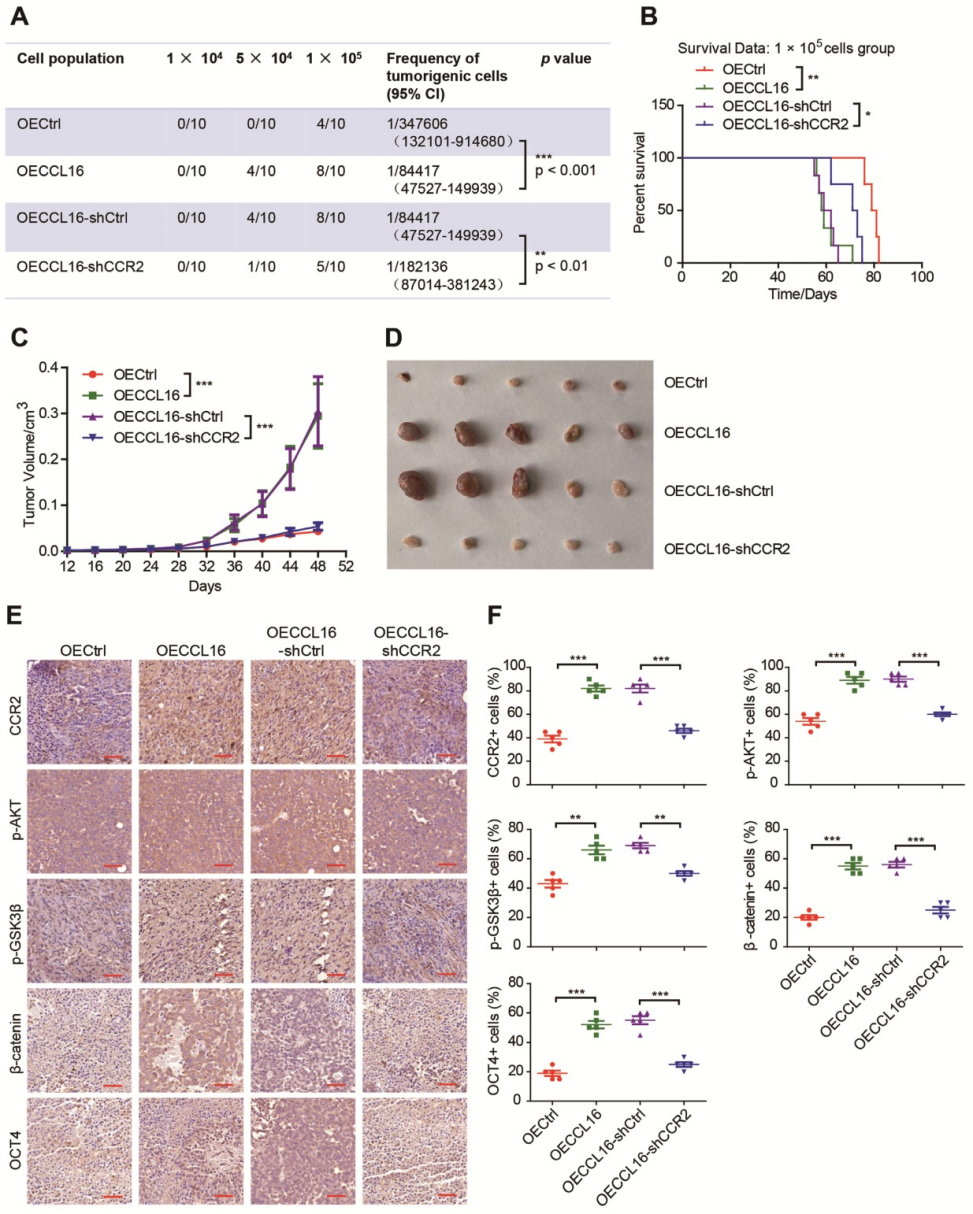
** CCR2 was indispensible for CCL16 mediated down-stream signaling activation and CSC-like identity maintenance *in vivo.* A. Limited dilution xenograft assay.** This figure shows that tumor initiation increases in OECCL16 (CCL16 ectopic expression) versus OECtrl (control) 1X10^5^ MDA-MB-231 cells xenografted in NOD/SCID mice. In addition, this increase in tumor initiation is reversed in OECCL16-shCCR2 (CCR2 knockdown) versus OECCL16-shCtrl (control) MDA-MB-231 cells xenografted in NOD/SCID mice. Subcutaneous tumors were harvested on the 28th-day post grafting (n = 10). CI means confidence interval. **B. Survival curve of mice in 1 × 10^5^ cell group.** This figure shows mouse survival time decreases in OECCL16 (CCL16 ectopic expression) versus OECtrl (control) MDA-MB-231 cells xenografted in NOD/SCID mice. In addition, this decrease in mouse survival time is reversed in OECCL16-shCCR2 (CCR2 knockdown) versus OECCL16-shCtrl (control) MDA-MB-231 cells xenografted in NOD/SCID mice. **C. & D. Tumor growth curve and representative dissected tumors.** These figures show that both tumor growth and tumor volume increases in OECCL16 (CCL16 ectopic expression) versus OECtrl (control) MDA-MB-231 cells xenografted in NOD/SCID mice. These increases in tumor growth and tumor volume were reversed in OECCL16-shCCR2 (CCR2 knockdown) versus OECCL16-shCtrl (control) MDA-MB-231 cells xenografted in NOD/SCID mice. **E. & F. Immunohistochemistry and quantification.** These figures show that immunostaining levels of CCR2, p-AKT, p-GSK3β, β-catenin, and OCT4 increase in OECCL16 (CCL16 ectopic expression) versus OECtrl (control) MDA-MB-231 cells xenografted in NOD/SCID mice. In addition, this increase in immunostaining is reversed in OECCL16-shCCR2 (CCR2 knockdown) versus OECCL16-shCtrl (control) MDA-MB-231 cells xenografted in NOD/SCID mice. 40 × mag; Scale bars: 50 μm

Since CCR2 contributes to CCL16-mediated signaling activation and CSC-like identity maintenance *in vitro*, we next explored whether this result could be substantiated *in vivo* using a limited dilution xenograft assay in NOD/SCID mice. We found that mice inoculated with 1 × 10^4^ CCL16-overexpressing MDA-MB-231 cells demonstrated a dramatically increased incidence of tumor initiation versus mice inoculated with control cells and that CCR2 knockdown prevented this increased tumor incidence (**Figure 7A**). Moreover, the survival data of mice inoculated with 1 × 10^5^ cells show that CCL16 overexpression shortened mouse survival time versus OECtrl and that CCR2 knockdown prevented this shortening (**Figure 7B**). In addition, CCL16 ectopic expression significantly stimulated tumor growth and tumor volume, which was prevented by CCR2 knockdown (**Figure 7C-D**). We also observed that CCL16 overexpression elevated the immunostaining of CCR2, p-AKT, p-GSK3β, and OCT4 in MDA-MB-231 cells, which was prevented by CCR2 knockdown (**Figure 7E-F**). Thus, our results indicate that CCR2 plays an indispensable role in CCL16-mediated p-AKT/GSK3β/β-catenin/OCT4 signaling activation and CSC-like identity maintenance *in vivo*.

### XAV939 inhibits CCL16-mediated β-catenin/OCT4 expression *in vitro* and breast cancer progression *in vivo*

**Figure 8:**
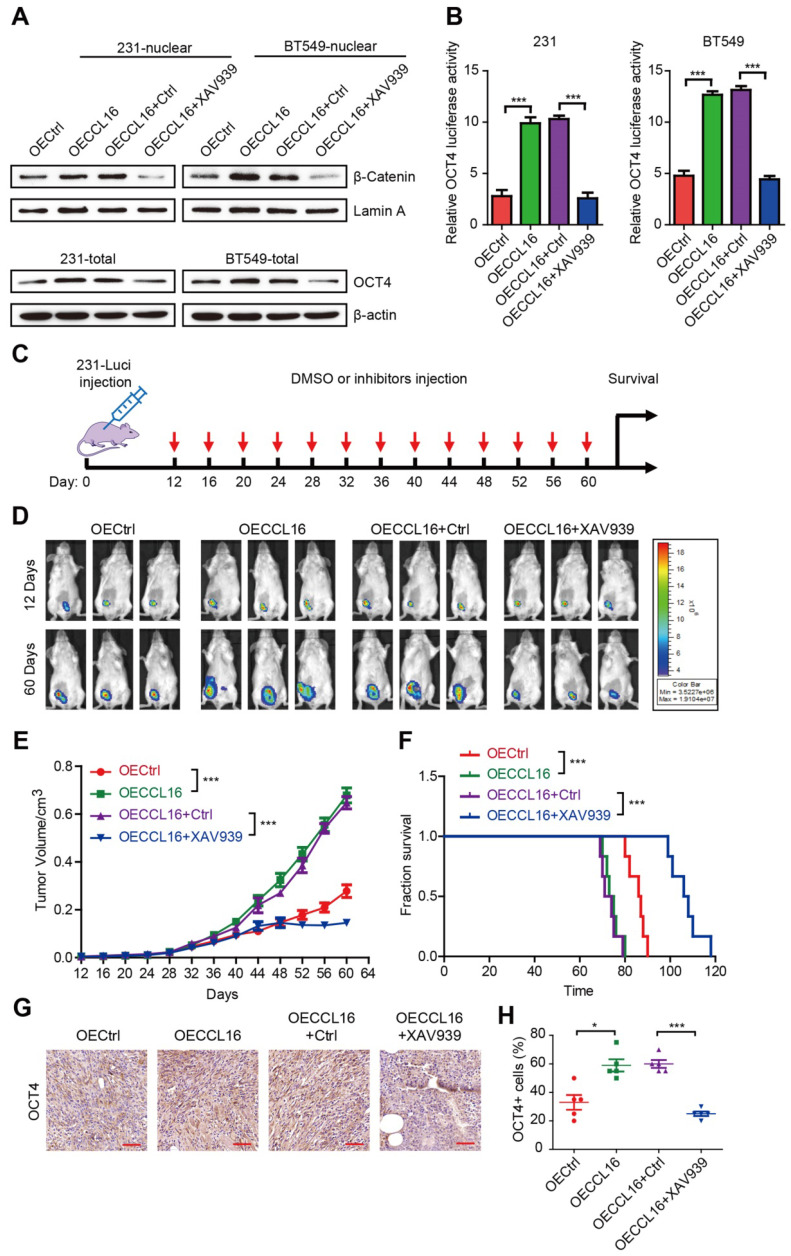
** XAV939 inhibits CCL16 mediated β-catenin/OCT4 expression *in vitro* and breast cancer progression *in vivo*. A. Western blot analysis.** This figure shows that expression of β-catenin in the nucleus increases in OECCL16 (CCL16 ectopic expression) versus OECtrl (control) MDA-MB-231 and BT549 cells. In addition, this increase in β-catenin nuclear expression is reversed in OECCL16 + XAV939 versus OECCL16+Ctrl MDA-MB-231 and BT549 cells. **B. Dual-luciferase assay**. This figure shows that OCT4 promoter activity increases in OECCL16 (CCL16 ectopic expression) versus OECtrl (control) MDA-MB-231 and BT549 cells. In addition, this increase in OCT4 promoter activity is reversed in OECCL16 + XAV939 versus OECCL16 + Ctrl MDA-MB-231 and BT549 cells. **C. Schematic timeline of the *in vivo* model involving implantation of MDA-MB-231-luci cells into NOD/SCID mice**. **D. & E. Representative MDA-MB-231-luciferase tumor images and tumor growth graph.** These figures show that tumor growth and progression increase in OECCL16 (CCL16 ectopic expression) versus OECtrl (control) MDA-MB-231-luci cells implanted into NOD/SCID mice. In addition, these figures show that tumor growth and progression are blocked in OECCL16 + XAV939 versus OECCL16 + Ctrl MDA-MB-231-luci cells implanted into NOD/SCID mice. **F. Survival curve of NOD/SCID mice implanted with MDA-MB-231-luci cells.** This figure shows that survival time decreases in NOD/SCID mice that were implanted with OECCL16 (CCL16 ectopic expression) versus OECtrl (control) MDA-MB-231-luci cells. In addition, survival time increases in NOD/SCID mice that were implanted with OECCL16 + XAV939 versus OECCL16 + Ctrl luci-MDA-MB-231 cells. **G. & H. Representative immunohistochemistry and quantification.** These figures show that OCT4 immunostaining increases in OECCL16 (CCL16 ectopic expression) versus OECtrl (control) MDA-MB-231-luci cells implanted into NOD/SCID mice. In addition, the increase in OCT4 immunostaining is blocked in OECCL16 + XAV939 versus OECCL16 + Ctrl MDA-MB-231-luci cells implanted into NOD/SCID mice. N = 4; Scale bar: 50 μm

To understand the role of β-catenin nuclear translocation in CCL16-mediated CSC-like identity maintenance *in vitro*, we used XAV939 (a β-catenin inhibitor) to suppress β-catenin nuclear translocation in MDA-MB-231 and BT549 cells and then studied its effects using various techniques. Using Western blot, we showed that CCL16 ectopic expression increased β-catenin nuclear translocation and that XAV939 blocked this increase (**Figure 8A**, upper panel). Using flow cytometry, we also found that CCL16 ectopic expression increased the ALDH+ sub-population, side population, and sphere formation, which was blocked by XAV939 (**Figure S4A-F**). In addition, using Western blot, we found that CCL16 ectopic expression increased the expression of OCT4 (a target gene of β-catenin) and that XAV939 blocked this increase (**Figure 8A**, lower panel). Finally, using a dual-luciferase assay, we observed that CCL16 ectopic expression increased OCT promoter activity and that XAV939 blocked this increase (**Figure 8B**).

To evaluate the role of XAV939 on breast cancer progression and as a therapeutic strategy, we performed xenograft experiments using MDA-MB-231-luci stable cell lines in NOD/SCID mice. NOD/SCID mice were inoculated with various MDA-MB-231-luci stable cell lines and then treated with XAV939 every 4 days beginning 12 days after inoculation (**Figure 8C**). Results from a luciferase assay show that XAV939 decreased CCL16-promoted tumor growth and tumor progression *in vivo* (**Figure 8D-E**). Furthermore, XAV939 prolonged mouse survival versus DMSO Ctrl (**Figure 8F**). In addition, using immunohistochemistry, we found that XAV939 decreased CCL16-induced OCT4 expression (**Figure 8G-H**). These findings suggest that, as a therapeutic strategy, XAV939 inhibits CCL16-mediated β-catenin/OCT4 expression *in vitro* and breast cancer progression *in vivo*.

### CCL16 expression is upregulated by IL10/STAT3 signaling *in vitro* and *in vivo*

**Figure 9:**
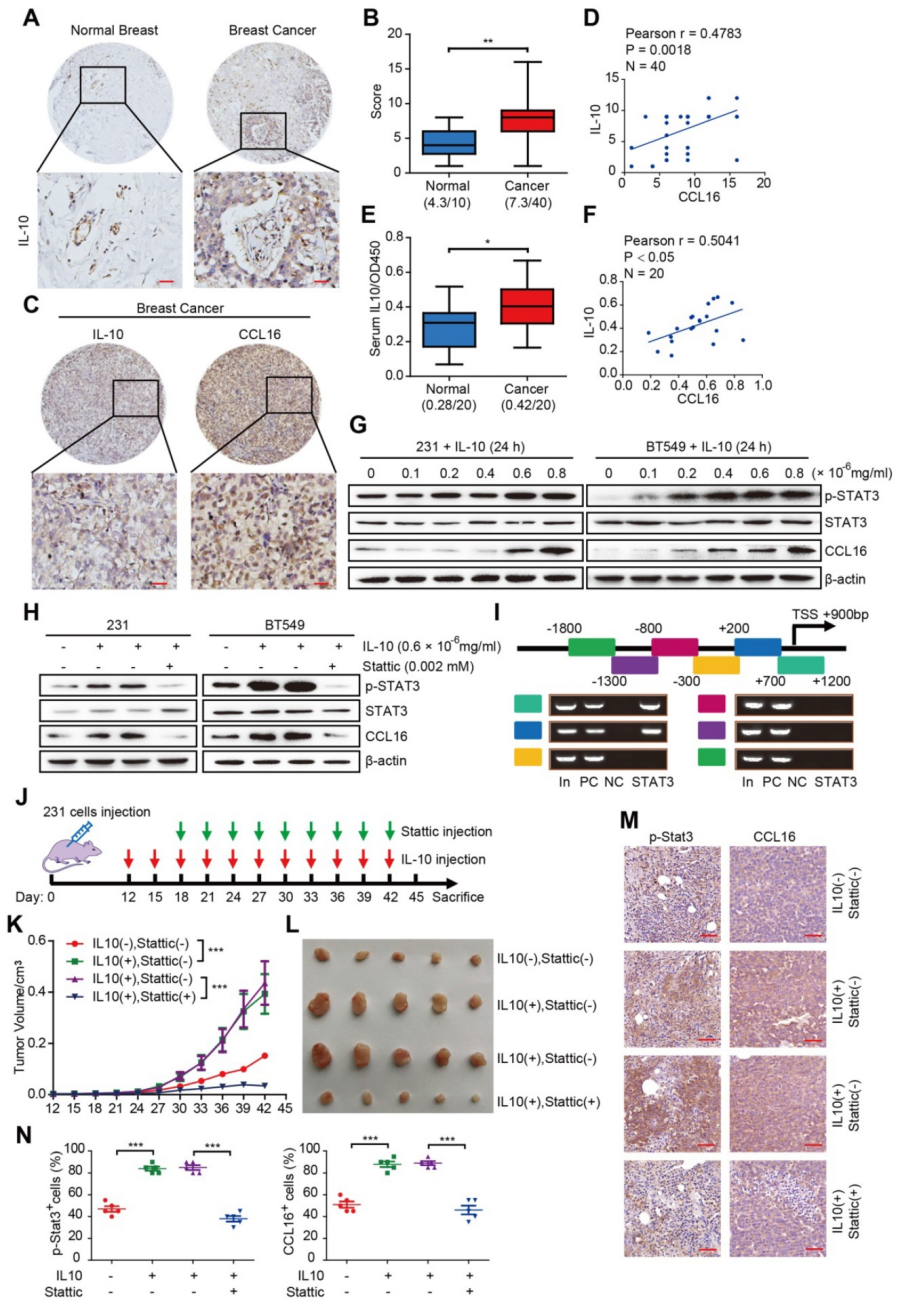
** CCL16 expression is up-regulated by IL10/STAT3 signaling *in vitro* and *in vivo.* A. Representative immunohistochemistry of IL10 in a human breast cancer tissue array.** This figure shows qualitatively that IL10 immunostaining increases in breast cancer tissue versus normal breast tissue. Scale bar: 50 μm **B.** Quantification of IL10 immunohistochemistry in a human breast cancer tissue array. This figure shows quantitatively that IL10 immunostaining increases in breast cancer tissue versus normal breast tissue. **C. & D. Representative immunohistochemistry of CCL16 and IL10 and correlation graphs in a human breast cancer tissue array.** These figures show that CCL16 and IL10 immunostaining correlate positively in breast cancer tissue. Scale bar: 50 μm **E. Measurement of serum IL10 in 20 pairs of breast cancer patients and normal patients.** This figure shows that serum IL10 levels significantly increase in breast cancer patient serum versus normal patient serum. **F. Correlation graph of CCL16 and IL10 in the serum of breast cancer patients.** This figure shows that serum CCL15 and serum IL10 correlate positively in the serum of breast cancer patients. **G. Western blot analysis.** This figure shows that IL10 induces p-STAT3 and CCL16 expression both of which are downstream targets of IL10 in MDA-MB-231 and BT549 cells. **H. Western blot analysis.** This figure shows that the expression of p-STAT3 and CCL16 increases in IL10-treated MDA-MB-231 and BT549 cells. In addition, this increase in p-STAT3 and CCL16 expression is blocked in Stattic-treated MDA-MB-231 and BT549 cells. **I. CHIP-PCR analysis.** This figure shows that p-STAT3 binding sites on the CCL16 promoter gene were located between + 0.2 kb to + 1.2 bp. **J. Schematic timeline of the *in vivo* model involving implantation of MDA-MB-231 cells into NOD/SCID mice. K. & L. Tumor growth curve and representative dissected tumors.** These figures show that tumor growth and tumor volume increase in IL10-treated versus untreated control MDA-MB-231 cells xenografted in NOD/SCID mice. In addition, these increases in tumor growth and tumor volume were blocked in Stattic-treated versus untreated control MDA-MB-231 cells xenografted in NOD/SCID mice. **M. & N. Representative immunohistochemistry and quantification.** These figures show that immunostaining of p-STAT3 and CCL16 increases in IL10-treated versus untreated control MDA-MB-231 cells xenografted in NOD/SCID mice. In addition, the increased immunostaining of p-STAT3 and CCL16 was blocked in Stattic-treated versus untreated control MDA-MB-231 cells xenografted in NOD/SCID mice. 40 × mag; n = 4; Scale bar: 50 μm

Having identified the functional and mechanistic roles of CCL16 in CSC-like identity maintenance, we next explored the reason why CCL16 upregulation occurs in breast cancer. Previous reports have indicated that CCL16 upregulation is linked to IL10 in monocytes and inflammatory cells 9, 10. In this regard, we first studied IL10 expression using immunohistochemistry in breast cancer tissue microarrays containing 50 samples (40 breast cancer tissues, 10 normal breast tissues). We observed increased IL10 immunostaining in breast cancer tissues versus normal breast tissue (**Figure 9A-B**). We next studied IL10 and CCL16 expressions using immunohistochemistry in breast cancer tissues. We observed a positive correlation between IL10 and CCL16 immunostaining in breast cancer tissues (**Figure 9C-D**). In addition, we measured serum IL10 in serum from patients with breast cancer and healthy subjects. We found significantly elevated serum IL10 in patients with breast cancer versus healthy subjects (**Figure 9E**). Moreover, serum IL10 shows a positive correlation with serum CCL16 in patients with breast cancer (**Figure 9F**).

To further elucidate the role of IL10 in CCL16 upregulation, we treated MDA-MB-231 and BT549 cells with various concentrations of human recombinant IL10 for 24 h and then measured the expression levels of p-STAT3 and CCL16, which are downstream targets of IL10. We found that 0.6 × 10^-6^ mg/mL IL10 induced STAT3 phosphorylation and CCL16 expression (**Figure 9G**). This finding suggests that IL10 mediates p-STAT3 nuclear translocation, which targets the CCL16 promoter and thereby regulates CCL16 expression. In order to pursue this line of thinking, we treated MDA-MB-231 and BT549 cells with 0.002 mM Stattic and 0.6 × 10^-6^ mg/mL IL10 for 24 h and then measured CCL16 expression. We observed that IL10 treatment increased expression of p-STAT3 and CCL16, which was blocked by Stattic treatment (**Figure 9H**).

To better understand how p-STAT3 regulates the CCL16 promoter gene, we first predicted the CCL16 promoter gene region (mainly located in the -3 kb to +2 kb region) using software and then performed a ChIP-PCR assay to scan the human CCL16 promoter gene from -1.8 kb to +1.2 kb for p-STAT3 binding sites. We found p-STAT3 binding sites located between +0.2 kb and +1.2 kb (**Figure 9I**). The above findings collectively suggest that IL10 mediates STAT3 phosphorylation, STAT3 activation, and STAT3 binding to the CCL16 promoter gene, which then regulates CCL16 expression *in vitro*.

To validate the above *in vitro* results, we performed xenograft experiments using MDA-MB-231 cells in NOD/SCID mice. NOD/SCID mice were inoculated with MDA-MB-231 cells and then treated with IL10 every 3 days beginning 12 days after inoculation and also treated with Stattic every 3 days beginning 18 days after inoculation (**Figure 9J**). Our findings demonstrate that inhibition of p-STAT3 signaling by Stattic decreased IL10-promoted tumor growth and tumor volume (**Figure 9K-L**). In addition, Stattic blocked IL10-induced p-STAT3 and CCL16 immunostaining in xenograft tumors (**Figure 9M-N**). In summary, our findings indicate that CCL16 expression is up-regulated by IL10/STAT3 signaling in breast cancer *in vitro* and *in vivo*.

### Proposed model of CCL16 in breast cancer CSC-like identity maintenance

**Figure 10:**
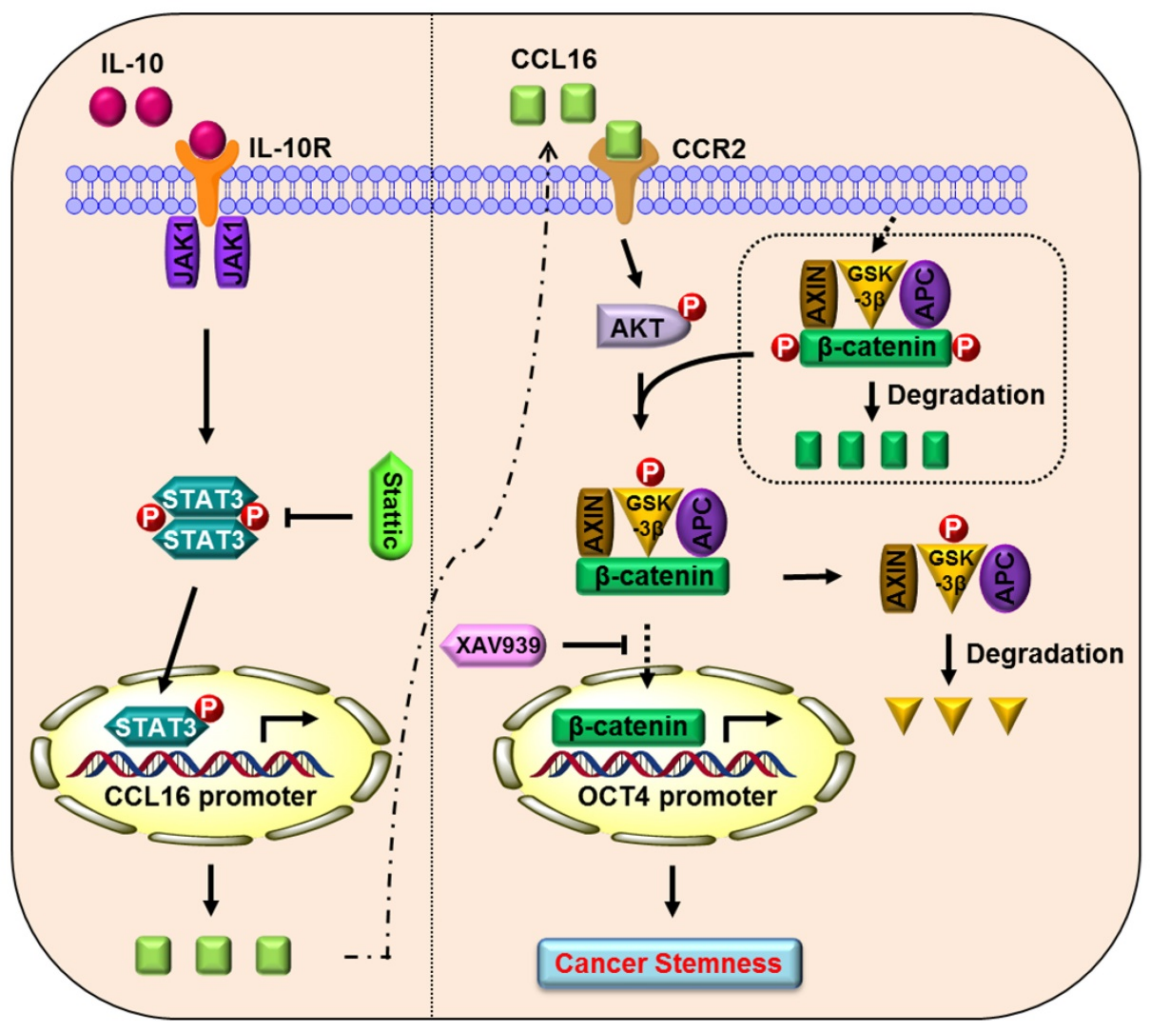
Proposed model of CCL16 in breast cancer CSC-like identity maintenance

Based on the totality of our findings, we propose the following model (**Figure 10**): CCL16 (a potential cancer stemness gene) binds to the CCR2 receptor and activates p-AKT/GSK3β signaling, which enhances β-catenin stability and promotes β-catenin nuclear translocation. β-catenin then binds to the OCT4 promoter gene, which mediates breast cancer CSC-like identity maintenance. XAV939 (a β-catenin inhibitor) blocks β-catenin nuclear translocation and thereby abates CCL16-mediated breast cancer CSC-like identity maintenance and breast cancer progression. In addition, IL10 (an upstream signal of CCL16) binds to the IL10 receptor and phosphorylates STAT3. The phosphorylated and activated STAT3 translocates into the nucleus, binds the CCL16 promoter, and facilitates CCL16 expression. Stattic (a STAT3 phosphorylation inhibitor) blocks STAT3 phosphorylation and activation and thereby reduces CCL16 expression. The reduced CCL16 expression abates CCL16-mediated breast cancer CSC-like identity maintenance and breast cancer progression.

## Discussion

CSCs are the chief culprits in tumor initiation, tumor malignancy, and tumor recurrence. CSCs depend on a unique tumor microenvironment (or niche) in order to maintain their stemness properties 26-30. Various clinical therapies target highly tumorigenic CSCs; but unfortunately, CSCs demonstrate a resistance to conventional chemotherapy and radiotherapy. Moreover, tumor-associated inflammation factors within the tumor niche promote cancer cell stemness and the resultant tumor initiation and malignancy. Our previous work presented a high-throughput siRNA screening platform that identifies inflammation genes that regulate cancer cell stemness. We identified several novel candidate genes (e.g., ICAM3, CCL16, PDE3A, and PRTN3) and showed that ICAM3 mediates cancer cell stemness as well as cancer-related inflammation via Src/PI3K/AKT signaling 18. In addition, we showed that the PDE3A gene maintains cancer cell stemness and may therefore serve as a target in breast cancer therapy 22. In this study, we observed that CCL16 (a potential cancer stemness gene) is overexpressed in breast tumors and significantly correlates with clinical progression. We found that CCL16 drives cancer cell stemness in MDA-MB-231 and BT549 breast cancer cells *in vitro* and in a xenograft NOD/SCID mouse model. Moreover, we explored the underlying mechanism and showed that CCL16 selects only the CCR2 receptor (no other CCR receptors) and activates the p-AKT/GSK3β signaling pathway. The p-AKT/GSK3β signaling pathway facilitates β-catenin nuclear translocation, OCT4 promoter binding, and OCT4 expression, which leads to enhanced breast cancer cell stemness. These findings support our screening results and suggest a novel therapeutic target for breast cancer.

CCL16 belongs to the CC chemokine family and plays a role in immunity and inflammation 31. A previous report indicated that CCL16 displays chemotactic activity for lymphocytes and monocytes but not for neutrophils 11. In addition, previous reports indicated that CCL16 also suppresses myeloid activity and myeloid progenitor cell proliferation. There is general agreement concerning the role of CCL16 in immunity and inflammation. However, the role of CCL16 in cancer remains uncertain, which led to our present study. In this regard, a previous report indicated that CCL16 mediates the recruitment of immune cells and induces anti-tumor immunity 28. Furthermore, bioinformatics studies have reported that CCL16 is a prognostic biomarker for triple-negative breast cancer and lung cancer metastasis 12. CCL16 also activates the angiogenic process in vascular endothelial cells via CCR1 and shows an elevated expression in lymphoid neo-organogenesis of breast cancer 32. In general, the existing knowledge about CCL16 was derived from big data analysis that looked at CCL16 mRNA expression levels. However, investigations into CCL16 protein expression in breast cancer tissues have been limited at best. In this study, we found overexpression of CCL16 protein in breast tumor tissue using tissue microarrays and in serum using ELISA. We also observed that CCL16 expression correlates with breast tumor grade and breast cancer progression. However, the functional role of CCL16 in other cancer types still remains an area for further study.

CCR2 is a G protein-linked receptor and the key receptor for the ligands CCL2, CCL7, CCL8, CCL13, and CCL16. In general, receptor-ligand binding leads to activation of various intracellular signaling pathways that mediate chemotactic responses. CCR2 expression occurs in macrophages 33, endothelial cells 34, fibroblasts 35, mesenchymal stem cells 36, and cancer cells 37, 38. Previous studies have shown that CCR2 expression correlates with prostate cancer progression/metastasis 39 and breast cancer progression 37. In addition, CCR2-CCL2 binding activates the Notch signaling pathway and thereby plays a role in breast cancer CSC-like identity maintenance 37. However, CCR2-CCL16 binding also plays a role in breast cancer CSC-like identity maintenance and may use different signaling pathways. In this study, we demonstrated that CCR2-CCL16 binding activates the GSK3β/β-catenin signaling pathway in order to maintain breast cancer CSC-like identity.

The canonical Wnt/β-catenin pathway causes β-catenin accumulation in the cytoplasm and β-catenin nuclear translocation. β-catenin accumulation is governed by a protein destruction complex composed of adenomatous polyposis coli (APC), casein kinase 1 (CK1), glycogen synthase kinase 3α/β (GSK-3α/β), and AXIN1 that tightly controls β-catenin levels in the cytoplasm via phosphorylation-mediated proteolysis 40, 41. Activation of the Wnt/β-catenin signaling pathway orchestrates various biological processes such as cell proliferation, differentiation, organogenesis, tissue regeneration, and tumorigenesis 42. However, different factors affect the stability of the protein destruction complex, which then mediates different biological processes. In our study, we demonstrated that CCL16 promotes GSK-3β phosphorylation and thereby prevents β-catenin destruction. This mediates nuclear translocation of β-catenin where it binds to the OCT4 promoter gene (i.e., a stemness gene); therefore, CCL16 plays a role in breast cancer CSC-like identity maintenance.

XAV939 is a cell-permeable small molecule that displays anti-tumor activity in colon, lung, liver, and breast cancers 43, 44. XAV939 binds specifically to the catalytic poly-ADP-ribose polymerase (PARP) domain of the tankyrase enzyme, which results in β-catenin destruction. Although some reports have indicated that XAV939 inhibits breast cancer progression 45, other reports have found that XAV939 shows little to no effect on triple-negative breast cancer. However, we found that XAV939 reduced β-catenin nuclear translocation and abolished CCL16-mediated breast cancer CSC-like identity maintenance *in vitro*. Moreover, we found that XAV939 slowed tumor progression in MDA-MB-231 breast cancer cells in a xenograft NOD/SCID mouse model. These findings may provide a valuable strategy in breast cancer therapy.

IL10/STAT3 is a classic signaling pathway that mediates an anti-inflammation response through the reduction of inflammatory cytokines (e.g., TNF-α, IL-6, and IL-1) by activated macrophages 46. IL10/STAT3 also promotes tumor proliferation and tumor metastasis via immunosuppression. IL10/STAT3-mediated immunosuppression of tumors is facilitated by tumor necrosis factor, IL-1, IL-12, and chemokines along with the down-regulation of CD80 and CD86 (surface co-stimulatory molecules) 47. IL10 stimulation up-regulates CCL16, however the mechanism of action remains unclear. In this study, we found that IL10 mediates STAT3 activation, which then binds to the CCL16 promoter gene and enhances CCL16 expression. In addition, we showed that Stattic blocks CCL16 expression* in vitro* and limits tumor progression *in vivo*.

In conclusion, we discovered that CCL16 drives stem-cell like properties in MDA-MB-231 and BT549 breast cancer cells *in vitro* and in a xenograft NOD/SCID mouse model. We demonstrated that CCL16 binds specifically to the CCR2 receptor and activates the p-AKT/GSK3β/β-catenin signaling pathway. This pathway activation promotes OCT4 expression and plays a role in breast cancer CSC-like identity maintenance. We also demonstrated that IL10 mediates STAT3 activation and thereby enhances CCL16 expression* in vitro* and *in vivo*. Finally, we take the position that CCL16 may serve as a novel therapeutic target in breast cancer treatment.

## Supplementary Material

Supplementary figures and tables.
