# CCL16 maintains stem cell-like properties in breast cancer by activating CCR2/GSK3β/β-catenin/OCT4 axis: Erratum

**DOI:** 10.7150/thno.62534

**Published:** 2021-05-15

**Authors:** Wenzhi Shen, Xiaoyuan Zhang, Jiaping Tang, Zhixin Zhang, Renle Du, Dehong Luo, Xiaoran Liu, Yong Xia, Yanping Li, Shanshan Wang, Siyuan Yan, Wancai Yang, Rong Xiang, Na Luo, Yunping Luo, Jianjun Li

**Affiliations:** 1 Dept. of Pathology and Institute of Precision Medicine, Jining Medical University, Jining 272067, China.; 2 Institute of Breast Research, Jining Medical University, Jining 272067, China.; 3 Dept. of Anatomy and Histology, School of Medicine, Nankai University, Tianjin 300071, China.; 4 Dept. of Gastrointestinal Surgery, Affiliated Hospital of Jining Medical University, Jining 272029, China.; 5 Dept. of Immunology, School of Medicine, Nankai University, Tianjin 300071, China.; 6 The First People's Hospital of Zunyi, Zunyi, 563002, China.; 7 Dept. of Immunology, Institute of Basic Medical Science, Chinese Academy of Medical Science, School of Basic Medicine Peking Union Medical College, Beijing, 100005, China.

In our article 1, there were misplaced images in Figure 7E and Figure 8D, respectively. The corrected version is provided here:

The correction made in this erratum does not affect the original conclusions. The authors apologize for any inconvenience or misunderstanding that this error may have caused.

## Figures and Tables

**Figure 7:**
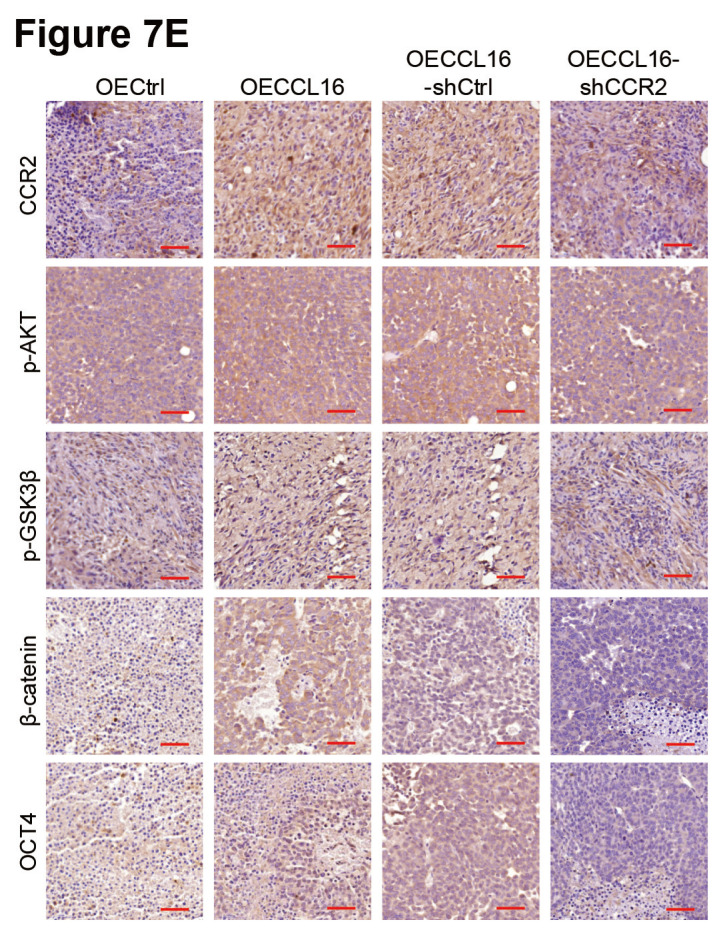
** E**. Immunohistochemistry staining of CCR2, p-AKT, p-GSK3β, β-catenin and OCT4 in each group tumors was performed. Representative images were shown. Scale bars: 50 μm.

**Figure 8:**
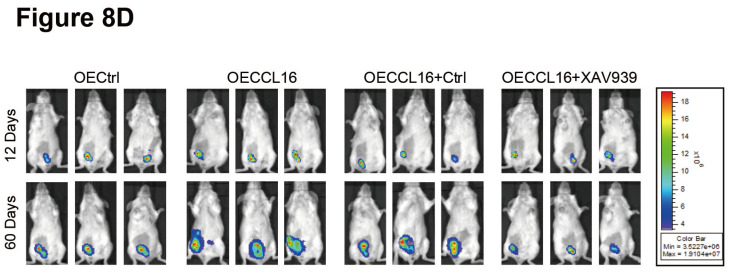
** D**. The representative luciferase images showing 231-luci tumors of each group on Day 12 (before XAV939 administration) and Day 60 (after XAV939 administration).
