# Supplementary material for: CCL16 maintains stem cell-like properties in breast cancer by activating CCR2/GSK3β/β-catenin/OCT4 axis
Source: Theranostics. 2021 Jan 1;11(5):2297–317. doi: 10.7150/thno.51000 (PMC7797668; doi:10.7150/thno.51000)
Supplement: Supplementary file 1 — Supplementary figures and tables. [file thnov11p2297s1.pdf]

Figure S1

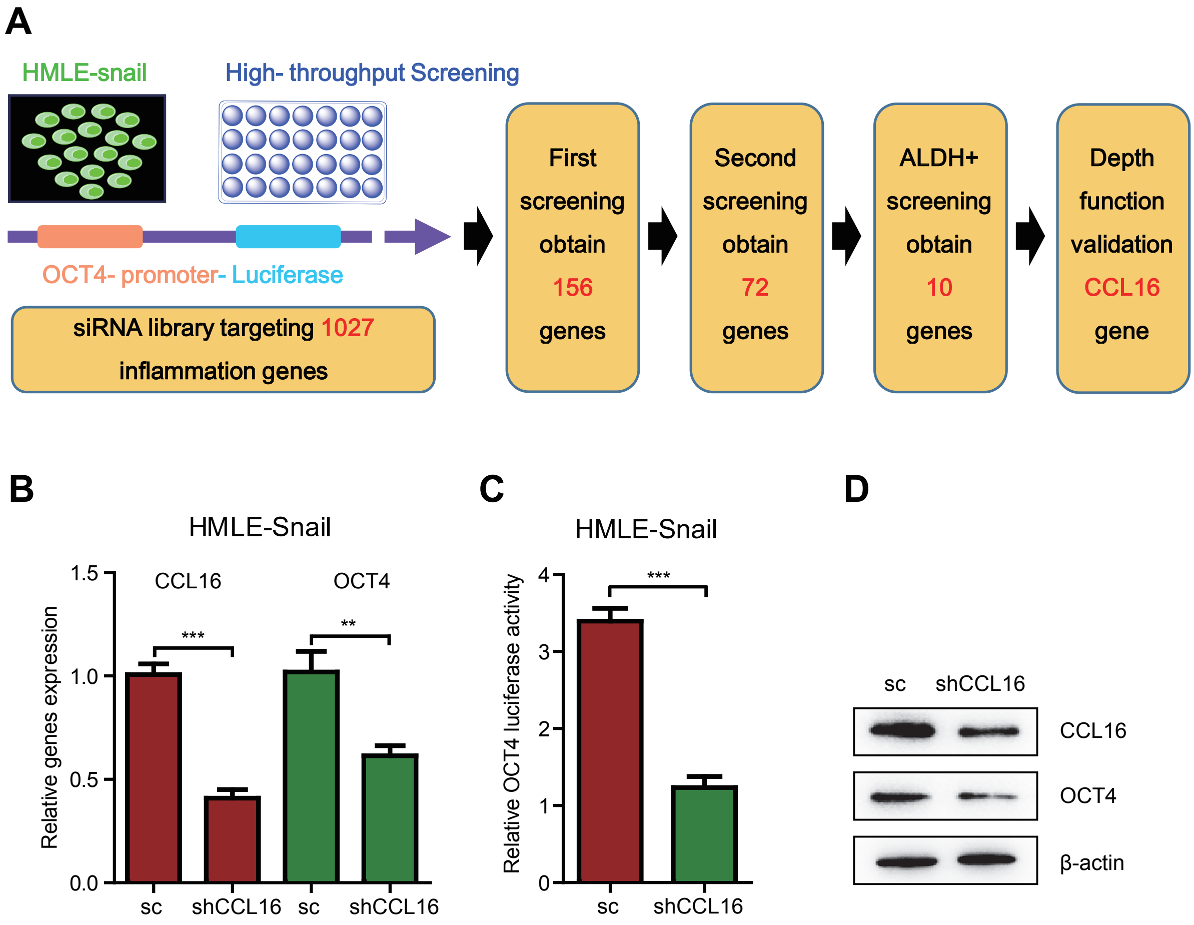

A
HMLE-snail
High- throughput Screening
OCT4- promoter- Luciferase
siRNA library targeting 1027 inflammation genes
First screening obtain 156 genes
Second screening obtain 72 genes
ALDH+ screening obtain 10 genes
Depth function validation CCL16 gene
B
HMLE-Snail
CCL16
OCT4
Relative genes expression
1.5
1.0
0.5
0.0
***
**
sc
shCCL16
sc
shCCL16
C
HMLE-Snail
Relative OCT4 luciferase activity
4
3
2
1
0
***
sc
shCCL16
D
sc
shCCL16
CCL16
OCT4
β-actin

Figure S1. Validation of the screening results of CCL16 in HMLE-snail cells.

A. Schematic representation and findings of the high-throughput siRNA screening platform. B. QPCR results. The figure shows that CCL16 and OCT4 mRNA expression decreases in shCCL16 (CCL16 knockdown) versus sc (control) HMLE-snail cells. **C. Dual-luciferase assay.** This figure shows that the activity of the OCT4 promoter decreases in shCCL16 (CCL16 knockdown) versus sc (control) HMLE-snail cells. **D. Western blot results.** This figure shows that the expression of CCL16 and OCT4 decreases in shCCL16 (CCL16 knockdown) versus sc (control) HMLE-snail cells.

Figure S2

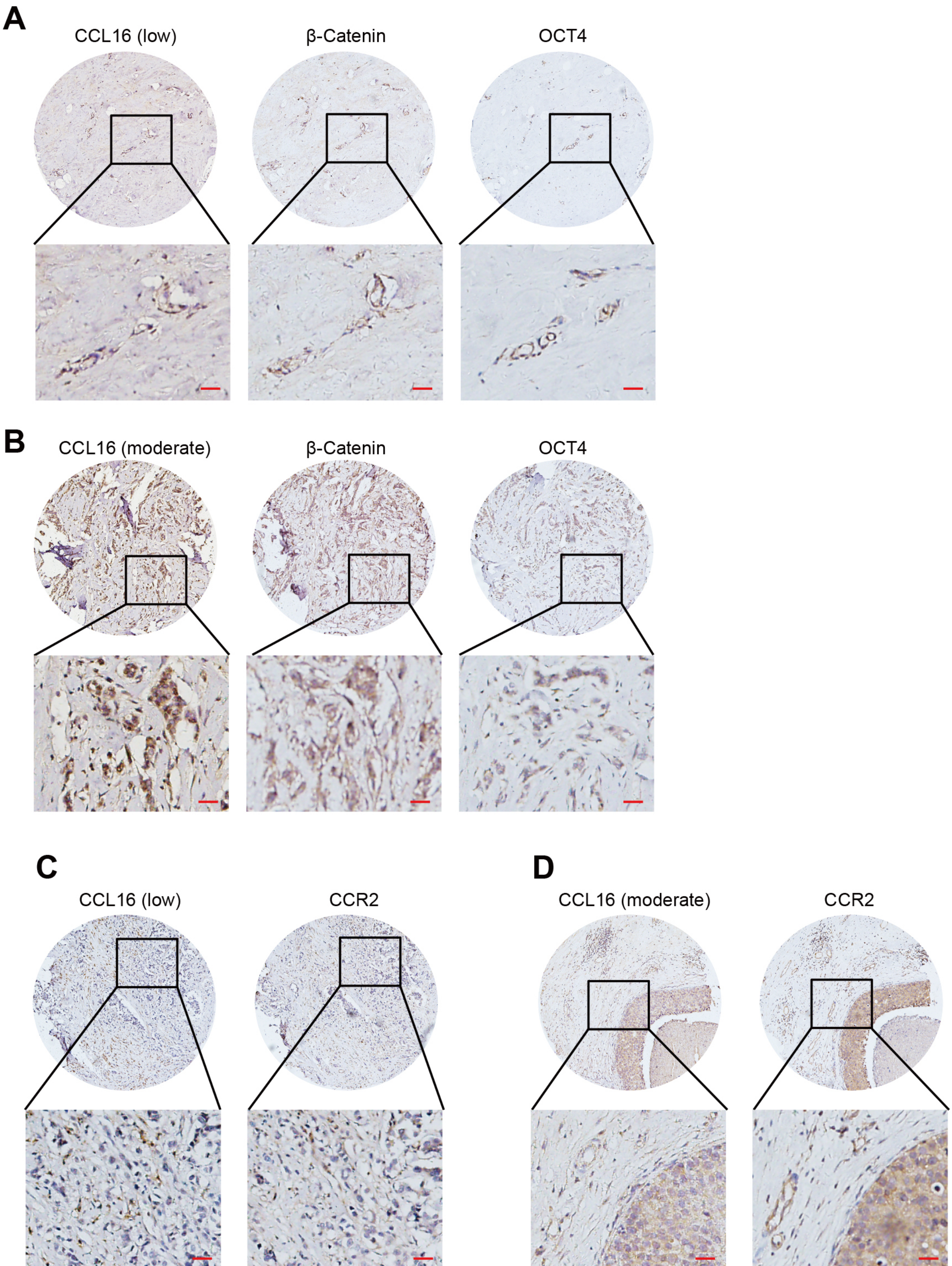
A
CCL16 (low)
β-Catenin
OCT4
B
CCL16 (moderate)
β-Catenin
OCT4
C
CCL16 (low)
CCR2
D
CCL16 (moderate)
CCR2

Figure S2. Representative immunohistochemistry and correlation graphs of a human breast cancer tissue array.

A. This figure shows the immunostaining of **low** CCL16, β-catenin, and OCT4 in human breast cancer tissue. **B.** This figure shows the immunostaining of **moderate** CCL16, β-catenin, and OCT4 in human breast cancer tissue. **C.** This figure shows the immunostaining of **low** CCL16 and CCR2 in human breast cancer tissue. **D.** This figure shows the immunostaining of **moderate** CCL16 and CCR2 in human breast cancer tissue.

Figure S3

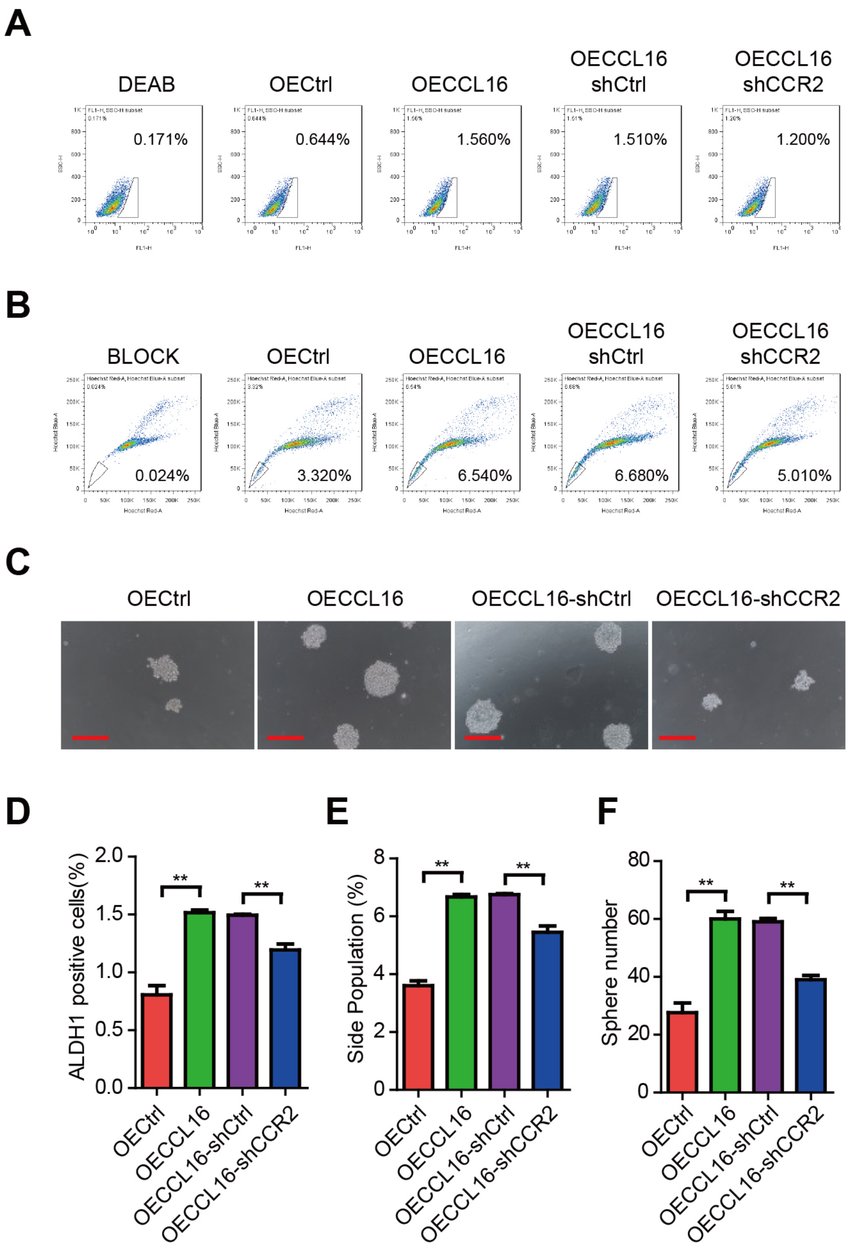
A
DEAB
OECtrl
OECCL16
OECCL16 shCtrl
OECCL16 shCCR2
0.171%
0.644%
1.560%
1.510%
1.200%
B
BLOCK
OECtrl
OECCL16
OECCL16 shCtrl
OECCL16 shCCR2
0.024%
3.320%
6.540%
6.680%
5.010%
C
OECtrl
OECCL16
OECCL16-shCtrl
OECCL16-shCCR2
D
ALDH1 positive cells(%)
E
Side Population (%)
F
Sphere number
OECtrl
OECCL16
OECCL16-shCtrl
OECCL16-shCCR2

Figure S3. CCR2 was indispensable for CCL16 mediated CSC-like identity maintenance.

A & D. ALDH staining assay. This figure shows that the ALDH+ sub-population decreases in shCCR2 MDA-MB-231 cells versus shCtrl cells (controls). **B & E. Side population assay.** This figure shows that the side-population decreases in shCCR2 MDA-MB-231 cells versus shCtrl cells (controls). **C & F. Sphere formation assay.** This figure shows that sphere formation decreases in shCCR2 MDA-MB-231 cells versus shCtrl cells (controls).

Figure S4

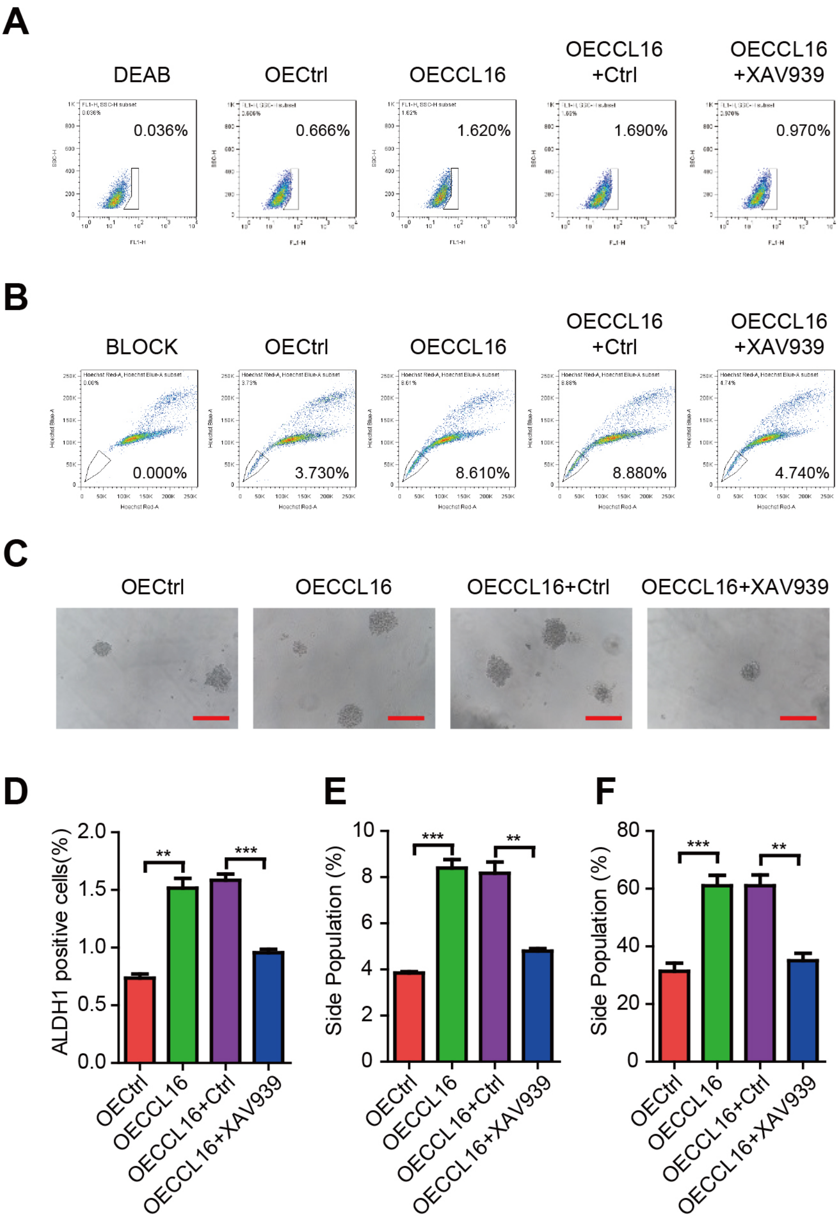
A
DEAB
OECtrl
OECCL16
OECCL16 +Ctrl
OECCL16 +XAV939
0.036%
0.666%
1.620%
1.690%
0.970%
B
BLOCK
OECtrl
OECCL16
OECCL16 +Ctrl
OECCL16 +XAV939
0.000%
3.730%
8.610%
8.880%
4.740%
C
OECtrl
OECCL16
OECCL16+Ctrl
OECCL16+XAV939
D
ALDH1 positive cells(%)
2.0
1.5
1.0
0.5
0.0
**
***
OECtrl
OECCL16
OECCL16+Ctrl
OECCL16+XAV939
E
Side Population (%)
10
8
6
4
2
0
***
**
OECtrl
OECCL16
OECCL16+Ctrl
OECCL16+XAV939
F
Side Population (%)
80
60
40
20
0
***
**
OECtrl
OECCL16
OECCL16+Ctrl
OECCL16+XAV939

Figure S4. β-catenin activation was indispensable for CCL16 mediated CSC-like identity maintenance.

A & D. ALDH staining assay. This figure shows that the ALDH+ sub-population decreases in XAV939 treated MDA-MB-231 cells versus Ctrl cells (controls). **B & E. Side population assay.** This figure shows that the side-population decreases in XAV939 treated MDA-MB-231 cells versus Ctrl cells (controls). **C & F. Sphere formation assay.** This figure shows that sphere formation decreases in XAV939 treated MDA-MB-231 cells versus Ctrl cells (controls).

Table S1.   Primer sequences

Name	Sequence
CCL16-RT-F	CTTATCATTACTTCGGCTTCTCGC
CCL16-RT-R	GGCCTTTCTGTATCCCACCACTA
CCL16-shRNA-1	AAAAGCCTGAAGTATTATGAGAAAGTTGGATCCAACTTTCTCATAATACTTCAGGC
CCL16-shRNA-2	AAAAGGGTCCAAGAGTACATCAAGGTTGGATCCAACCTTGATGTACTCTTGGACCC
shCtrl	AAAAGCTACACTATCGAGCAATTTTGGATCCAAAATTGCTCGATAGTGTAGC
FLAG-CCL16-F	GCTCTAGAGCCACCATGGATTACAAGGATGACGACGATAAGAGCCCGATGAAGGTCTCCGAGGCT
CCL16-R	CGACGCGTTCACTGGGAGTTGAGGAGC
NANOG-RT-F	TCTGGACACTGGCTGAATCCT
NANOG-RT-R	CGCTGATTAGGCTCCAACCAT
SOX2-RT-F	GCCTGGGCGCCGAGTGGA
SOX2-RT-R	GGGCGAGCCGTTCATGTAGGTCTG
OCT4-RT-F	GCTCGAGAAGGATGTGGTCC
OCT4-RT-R	CGTTGTGCATAGTCGCTGCT
OCT4-CHIP-1-F	ACTGGTTCATGTGGGGAAGGT
OCT4-CHIP-1-R	GGCTGGGGCAGCCCAT

OCT4-CHIP-2-F	AGGGATGGGCTGCCCCA
OCT4-CHIP-2-R	GGAGGAGGCCGGGAGCG
OCT4-CHIP-3-F	CACCGCTCCCGGCCT
OCT4-CHIP-3-R	TCCAGACACTCTTACCTCAAATAGA
OCT4-CHIP-4-F	TTGAGGTAAGAGTGTCTGGAT
OCT4-CHIP-4-R	ACACACACCTTTATTATTACAGTG
OCT4-CHIP-5-F	GTAATAATAAAGGTGTGTGTGAA
OCT4-CHIP-5-R	AGTCCCTGCTGCCCA
OCT4-CHIP-6-F	GAAAATGGGCAGCAGGGA
OCT4-CHIP-6-R	GTGGCCAGCTGTCTTCATCT
OCT4-CHIP-7-F	AGATGAAGACAGCTGGCCAC
OCT4-CHIP-7-R	CCCGAGCCTGGCAGAT
OCT4-CHIP-8-F	CTCAATCTGCCAGGCTCG
OCT4-CHIP-8-R	AACTCAGACATCTAATACCACGGTA
OCT4-CHIP-9-F	TGGTATTAGATGTCTGAGTTTTGGT
OCT4-CHIP-9-R	CTCTCAGCTCCTCAAATTTATTGA
OCT4-CHIP-10-F	TAAATTTGAGGAGCTGAGAGGGT
OCT4-CHIP-10-R	CCTCAGTGCAGGTCCCCC
CCL16-CHIP-1-F	TTCTCCATCCCCCAGCCTA
CCL16-CHIP-1-R	AGATCACGAGGTCAGGAGTTTG

CCL16-CHIP-2-F	GGAGCAGCTGGCAGGGA
CCL16-CHIP-2-R	GGGGACCATAGATTCCCAAG
CCL16-CHIP-3-F	CGCCTATCCACCCAGGTG
CCL16-CHIP-3-R	ACCTCCCTGCCAGCTGCT
CCL16-CHIP-4-F	GGAGCACAGACGCAGGTTGT
CCL16-CHIP-4-R	GTCACCTGGGTGGATAGGCG
CCL16-CHIP-5-F	AGGAAGGGCTTGTGGGC
CCL16-CHIP-5-R	ACAACCTGCGTCTGTGCTC
CCL16-CHIP-6-F	GCATGAAGACATTTTATCCAACC
CCL16-CHIP-6-R	GTGGCCCACAAGCCCTT

Table S2. Antibodies List

Antibody		Clone, Cat #	Vendor	City, State, Country
CCL16	Rabbit monoclonal	EPR4452(2), ab134917	Abcam	Hong Kong, China
OCT4	Rabbit polyclonal	ab19857	Abcam	Hong Kong, China
SOX2	Rabbit monoclonal	EPR3131, ab92494	Abcam	Hong Kong, China
NANOG	Rabbit polyclonal	Ab80892	Abcam	Hong Kong, China
β-catenin	Rabbit polyclonal	ab16051	Abcam	Hong Kong, China
ALDH1A1	Rabbit monoclonal	EP1933Y, ab52492	Abcam	Hong Kong, China
CCR1	Rabbit polyclonal	Ab19013	Abcam	Hong Kong, China
CCR5	Rabbit polyclonal	Ab65850	Abcam	Hong Kong, China
CCR8	Rabbit polyclonal	Ab63772	Abcam	Hong Kong, China
β-actin	Mouse monoclonal	sc-47778	Santa Cruz Biotechnology	Santa Cruz, CA, USA
p-GSK3β	Rabbit monoclonal	9323	Cell Signal Technology	Danvers, MA, USA
GSK3β	Rabbit monoclonal	12456	Cell Signal Technology	Danvers, MA, USA
LaminA	Mouse monoclonal	133A2	Cell Signal Technology	Danvers, MA, USA
CCR2	Rabbit monoclonal	D14H7	Cell Signal Technology	Danvers, MA, USA
p-AKT	Rabbit monoclonal	4060	Cell Signal Technology	Danvers, MA, USA
AKT	Rabbit monoclonal	4685	Cell Signal Technology	Danvers, MA, USA
FLAG	Rabbit monoclonal	14793	Cell Signal Technology	Danvers, MA, USA
p-STAT3	Rabbit monoclonal	9145	Cell Signal Technology	Danvers, MA, USA
STAT3	Rabbit monoclonal	12640	Cell Signal Technology	Danvers, MA, USA
